# HLA-II Alleles Influence Physical and Behavioral Responses to a Whey Allergen in a Transgenic Mouse Model of Cow's Milk Allergy

**DOI:** 10.3389/falgy.2022.870513

**Published:** 2022-04-14

**Authors:** Danielle L. Germundson, Suba Nookala, Nicholas A. Smith, Yassmine Warda, Kumi Nagamoto-Combs

**Affiliations:** ^1^Department of Pathology, Clinical and Translational Sciences Graduate Program, University of North Dakota School of Medicine & Health Sciences, Grand Forks, ND, United States; ^2^Department of Biomedical Sciences, University of North Dakota School of Medicine & Health Sciences, Grand Forks, ND, United States

**Keywords:** beta-lactoglobulin, cow's milk allergy, cytokines, HLA class II, IgE, spatial memory, splenocytes

## Abstract

The symptoms of food allergies vary significantly between individuals, likely due to genetic determinants. In humans, allergy development is initiated by antigen-presenting cells *via* class II human leukocyte antigen (HLA-II). The HLA-II gene is highly polymorphic, and its allelic variance is thought to influence the susceptibility of individuals to a particular allergen. However, whether antigen presentation by different HLA-II variants contributes to symptom variation is not clear. We hypothesized that HLA-II allelic variance affects symptom phenotypes, including immediate physical reactions and delayed behavioral changes, in individuals with food hypersensitivity. To test our hypothesis, male and female mice of three transgenic strains expressing an HLA-II variant, DR3, DR15, or DQ8, were used to establish a cow's milk allergy model. Mice were sensitized to a bovine whey allergen, β-lactoglobulin (BLG; Bos d 5), weekly for 5 weeks, followed by an acute oral allergen challenge. At 30 min post-challenge, BLG-sensitized DR3 mice showed moderate to severe anaphylaxis resulting in perioral redness, swelling, and death. In contrast, DQ8 and DR15 mice were generally asymptomatic. The production of allergen-specific immunoglobulins was also HLA- and sex-dependent. Both male and female DR3 and female DR15 mice significantly increased BLG-specific IgE production, while robust elevation in BLG-specific IgG1 was observed in sensitized DQ8 mice of both sexes and, to a lesser extent, in DR15 males. Furthermore, BLG-sensitized DR15 mice showed sex-specific behavior changes, with males exhibiting mobility changes and anxiety-like behavior and females showing spatial memory impairment. When splenocytes from transgenic mice were stimulated *in vitro* with BLG, phenotypes of immune cells were HLA- and sex-specific, further underscoring the influence of HLA-II on immune responses. Our results support that HLA-II alleles influence behavioral responses in addition to immune and physical reactions of food allergy, suggesting that certain HLA-II variants may predispose individuals to food-allergy-associated behavioral changes.

## Introduction

Food allergy is an increasingly prevalent atopic disorder, with an estimated 220 million individuals affected globally (1–4). Reactions to ingested allergens are diverse, ranging from life-threatening anaphylaxis characterized by the rapid development of urticaria, swelling, airway constriction, hypotension, and systemic shock (5), to less severe gastrointestinal and dermatological manifestations (6–8). In addition, many studies have found that food allergy is often associated with neuropsychiatric disorders such as anxiety (9–11), depression (10, 12), attention deficit hyperactivity disorder (10–12), tic disorders (13, 14), and autism spectrum disorder (15, 16), suggesting that the consequences of food allergy include widely variable behavioral abnormalities.

Multiple factors likely affect symptom heterogeneity, such as genetic background, intestinal microbiota, environment, and diet [see reviews (2, 17)]. Previously, we used a mouse model of non-anaphylactic cow's milk allergy (CMA) and demonstrated that sensitization of C57BL/6J and BALB/cJ strain mice to a bovine whey allergen, β-lactoglobulin (BLG: Bos d 5), produced strain- and sex-specific physical reactions and behavior changes in association with distinctly altered immune responses and microbiome (18). These findings underscored the significance of genetic background in allergen-induced symptom manifestations and biological responses. The C57BL/6J and BALB/cJ strains are considered to have distinct Th1- and Th2-biased immune responses (17, 19, 20), and the sex-dependent dichotomy in immune systems and symptomatic response to food allergy is well-recognized (21–23). Thus, intrinsic variations in immune components likely play a major role in determining symptom outcomes.

The class II major histocompatibility complex (MHC-II) is a highly polymorphic transmembrane molecule expressed by professional antigen-presenting cells (APCs), such as dendritic cells, macrophages, and B lymphocytes. Through its variable epitope-binding domain, it interacts with diverse peptides from extracellular antigens that have been phagocytosed and processed by the APCs. The antigen peptide is then transported to the cell surface and presented to T lymphocytes to activate the differentiation and proliferation of CD4^+^ T helper (Th) cells to promote subsequent antibody production *via* B cell differentiation (24). The human ortholog of MHC-II is encoded by the human leukocyte antigen class II (HLA-II) gene and expressed as HLA-DP, HLA-DR, or HLA-DQ α and β heterodimers (24).

Some allelic variants of HLA-II have been linked to specific diseases. For example, the associations of HLA-DR15 with multiple sclerosis (MS) (25–27) and HLA-DQ8 with type I diabetes (28, 29) have been well-established, and the haplotypes of these alleles are considered risk factors for the respective disorders. For food allergy, DR1-DQB1^*^05:01 and DR15-DQB1^*^0602 haplotypes were associated with CMA, particularly with greater humoral responses to BLG (30). Furthermore, bioinformatic analysis of the interactions between BLG epitopes and common HLA-DR/DQ molecules has suggested that DRB1^*^01:01, DQ7 and DQ8 increase the susceptibility to CMA, while DRB1^*^03:01, DRB1^*^04:04, DRB1^*^12:01, and DRB1^*^15:01 haplotypes are protective (31). Thus, the expression of particular HLA-II haplotypes likely influences the selectivity of antigen presentation and the subsequent chain of immune responses, affecting the development of hypersensitivity to BLG as well as the type and severity of associated CMA symptoms.

In the present study, we therefore hypothesized that allelic variations of HLA-II would produce distinct immune responses to BLG, leading to variant-specific physiological reactions and behavioral manifestations in individuals with CMA. To test our hypothesis, we sensitized transgenic mice carrying HLA-II alleles, DRB1^*^03:01 (DR3), DRB1^*^15:01 (DR15), or DQB1^*^0302 (DQ8), to BLG and assessed their immediate physical reactions to the allergen upon challenge and subsequent changes in their immune responses and behavioral changes. We also further examined the differing immune responses among the HLA strains by stimulating splenocytes from the transgenic mice *in vitro* to BLG.

## Methods

### Mice

Male and female transgenic mice expressing HLA-II alleles, DRB1^*^03:01 (DR3), DRB1^*^15:01 (DR15), or HLA-DQA1^*^0301 and DQB1^*^0302 (DQ8), were bred in-house in a specific pathogen-free room at the University of North Dakota. These transgenic mice have been shown to express functional HLA (32, 33), and the presence of the appropriate HLA-II transgenes was confirmed by PCR-based genotyping using allele-specific oligonucleotide primers as described previously (33, 34). Animals were housed under a 12-h light/12-h dark cycle and had *ad libitum* access to water and a whey-protein-free diet (Teklad 2018, Envigo Corporation, Indianapolis, IN, USA). All procedures were carried out in strict accordance with the Guide for the Care and Use of Laboratory Animals of the National Institutes of Health and approved by the University of North Dakota Institutional Animal Care and Use Committee.

### BLG Sensitization of HLA Transgenic Mice

**Figure 1:**
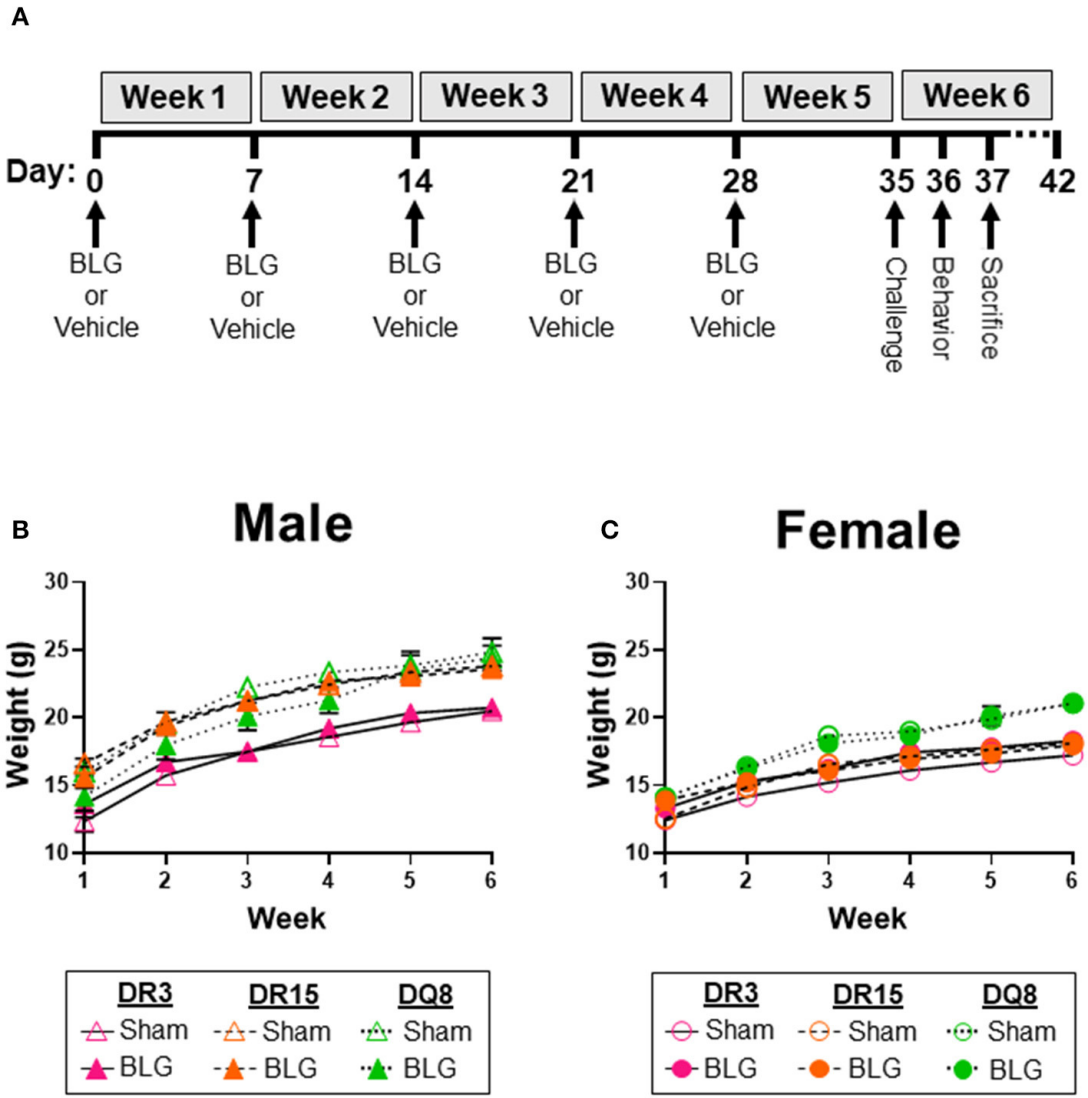
Experimental outline and weekly weight measurements of mice. Four-week-old HLA-II DR3, DR15, or DQ8 mice were subjected to a weekly intragastric administration of either vehicle (10 μg CT in carbonate buffer) or 1 mg BLG in the vehicle **(A)**. On Day 35, both treatment groups were given an oral challenge of 50 mg BLG in the vehicle, and their immediate physical responses were observed. Behavioral testing was performed on Day 36, and mice were sacrificed on Day 37. The weight of sham and BLG-sensitized mice was recorded weekly to measure their growth during the allergic sensitization **(B,C)**. Symbols represent group average ± SEM at each time point. Sham mice (open symbols); BLG mice (closed symbols). HLA strain: DR3 (pink with solid line); DR15 (orange with long dashed line); DQ8 (green with short dashed line) (*n* = 7–10 per treatment group).

BLG sensitization was carried out as previously described (18, 35). Briefly, 4-week-old genotyped transgenic mice were randomly assigned to sham or BLG-sensitization groups (*n* = 6–11). Once a week for 5 weeks, the BLG-sensitization group was intragastrically gavaged with 1 mg of purified BLG (Millipore Sigma, Burlington, MA, USA) in 200 μL sodium bicarbonate buffer (pH 9.0) with 10 μg cholera toxin (CT; List Biologicals, Campbell, CA, USA) as an adjuvant, while the sham group received only the CT-containing vehicle (Figure 1A). All mice were fasted for 2 h before and after sensitization. At the beginning of Week 6, both the sham and BLG-sensitized mice were orally challenged with 50 mg BLG (without CT) in 200 μL sodium bicarbonate buffer. At 30 min post-challenge, their body temperature was measured using a MicroTherma 2 T Handheld Thermometer with a RET-3 probe (Braintree Scientific, Inc., Braintree, MA, USA), and their physical symptoms were scored based on the scale by Li et al. (36).

### Behavior Analysis

#### Open Field Test (OFT)

General activity and anxiety-like behavior were evaluated using the open field test (OFT) 1 day after the BLG challenge. As described previously (18, 37), mice were placed individually in a plexiglass arena (San Diego Instruments, San Diego, CA, USA) and allowed to acclimate for 30 s before their activity was video recorded for 10 min with an overhead CCD digital video camera placed above the enclosure (C525 HD webcam, Logitech International, Newark, CA, USA). The arena was thoroughly cleaned between the testing of animals with disinfecting solution (Process NPD; STERIS, Mentor, OH, USA). Total time mobile, distance traveled, immobile episodes, and time in the center zone were quantified by the ANY-maze software (Stoelting, Wood Dale, IL, USA). As mice tend to become acclimated to the open field during the testing period (18), their behavior was analyzed for the first (0–4 min) and the second (5–10 min) portions separately to assess potential time-dependent effect during the 10-min recording session.

#### Cross Maze Test (CMT)

Spatial memory was evaluated using the cross maze test (CMT) as previously described (18). Individual mice were placed into a starting arm of the four-arm apparatus, and the patterns of their exploratory activity were video recorded for 12 min with a CCD digital video camera placed above the enclosure (Logitech International). The maze was thoroughly cleaned with Process NPD (STERIS) between tests. An observer blinded to the treatment and strains of the mice quantified the number and sequence of arm entries from the video recordings. A successful spontaneous alternation was defined as consecutive entries into each of the maze's arms without entering the same arm more than once. The percent of spontaneous alternations was calculated using the formula below (18, 38). Greater percentages of spontaneous alternations were considered to reflect better spatial memory.


$$\begin{array}{l} \text{\% Spontaneous alternations}=\frac{\#\;spontaneous\;alternations}{total\;arm\;entries\;-\;3}\times 100 \end{array}$$

### Tissue Sample Collection

Mice were euthanized by CO_2_-asphyxiation 2 days after the BLG challenge. Atrial blood was collected into microfuge tubes or EDTA-coated Microvette tubes (Sarstedt, Inc., Newton, NC, USA). The blood collected in microfuge tubes was centrifuged at 2,000 × *g* for 15 min at 4°C to collect the serum and stored at −80°C to determine BLG-specific IgG and IgE levels. Ten microliter of whole blood from the EDTA-coated tubes was thinly smeared onto a glass slide for later microscopic analysis. The remaining blood in the circulation was cleared by transcardial perfusion with phosphate-buffered saline (PBS; pH 7.4). The ileum was dissected from the rest of the small intestine, fixed in 4% paraformaldehyde (pH 7.4) for 24 h at 4°C, and equilibrated in 30% sucrose in PBS prior to sectioning.

### BLG-Specific IgE and IgG1 Enzyme-Linked Immunosorbent Assays (ELISAs)

Isotype-specific detection of immunoglobulins (Igs) was carried out as previously described (18, 39). Briefly, 8-well RIA strips (Corning, Inc., Corning, NY, USA) were coated with 2 μg/mL of sterile BLG solution in sodium carbonate/bicarbonate buffer (pH 9.5) overnight at 4°C. Wells were washed and blocked in an assay buffer containing 0.5% bovine serum albumin (BSA) in PBS. Serum samples were diluted to 1:40 in assay buffer and incubated with magnetic protein-G Dynabeads™ (Thermo Fisher, Waltham, MA, USA) to adsorb total IgG. The supernatant was collected for IgE ELISA, while bead-bound IgG was eluted with 50 mM glycine buffer (pH 2.8) and neutralized with 1 M tris buffer (pH 7.5) for IgG1 ELISA. Both sample preparations were incubated in the BLG-coated plates overnight at 4°C. Wells were washed thoroughly with the assay buffer and incubated with a secondary anti-mouse IgE (Thermo Fisher Scientific, Cat# 13-5992-82) or IgG1 (ThermoFisher Scientific, Cat# 13-4015-82) at 1:1,000 dilution. The amounts of IgE and IgG1 were quantified by the colorimetric detection of 3,3′,5,5′-tetramethylbenzidine (TMB) as the substrate after incubating with avidin-HRP (eBioscience, San Diego, CA, USA). The substrate reactions were terminated with 2 N H_2_SO_4_, and the plates were immediately read at 450 nm with a reference wavelength at 550 nm (BioTek Instruments, Winooski, VT, USA).

### Mast Cell Protease 1 (MCPT-1) ELISA

Mast cell activities were determined by the amounts of circulating MCPT-1 as described previously (40). The serum samples collected at the time of sacrifice were diluted 1:10 with the assay buffer, and 100 μL of the diluted samples were used to quantify MCPT-1 using the Mouse MCPT-1 Uncoated ELISA Kit (Thermo Fisher Scientific) according to the manufacturer's instructions.

### Blood Smear Staining and Differential Blood Counting

The blood smears from the anticoagulated whole blood samples were fixed in methanol for 10 min and allowed to dry. The slides were stained with Wright-Giemsa stain (Volu-Sol, Salt Lake City, UT, USA) according to the manufacturer's instructions and coverslipped with Permount mounting medium (Fisher Scientific, Hampton, NH, USA). Two blinded observers, a board-certified medical laboratory scientist (DG) and a pathology resident (YW), performed the 5-part differential blood counting and calculated the relative percentage of lymphocytes, neutrophils, monocytes, eosinophils, and basophils of 200 leukocytes.

### Ileum Hematoxylin and Eosin (H and E) Staining and Villi Measurement

Sucrose-equilibrated ileum samples were embedded in Tissue-Tek® O.C.T. Compound (Sakura Finetek USA, Inc, Torrance, CA, USA) and sectioned at 10 μm on a Leica CM1850 cryostat (Leica Biosystems, Buffalo Grove, IL, USA). Ileum sections were then stained with H&E and coverslipped at the University of North Dakota Histology Core Facility. Whole-slide images were captured using the Hamamatsu NanoZoomer 2.0HT Brightfield + Fluorescence Slide Scanning System (Hamamatsu Photonics, Bridgewater, NJ, USA). Using the measure tool on the NDP.view2 software (Hamamatsu Photonics), 5 villi per section were measured from the intestinal crypts to the apical tip of the villi. The villi measurements were averaged to calculate the villi length of each animal.

### Splenocyte Culture and *in vitro* BLG Stimulation

The spleens were excised from 3 to 4 months-old naïve male and female HLA-DR3, HLA-DR15, and HLA-DQ8 mice after euthanasia with CO_2_ asphyxiation and individually collected in ice-cold PBS (pH 7.4). Each spleen was mashed on a sterile 70-μm cell strainer placed over a 50-mL conical tube and rinsed through the strainer with PBS. Splenocytes were pelleted by centrifuging the resulting cell suspension at 400 × *g* for 5 min at 4°C and resuspended in red blood cell lysis buffer (BioLegend, San Diego, CA, USA) for 2 min at room temperature. Lysis was terminated by adding 30 mL PBS and removing the lysis buffer by centrifugation. The resulting cell pellets were resuspended in RPMI-1640 medium supplemented with 25 mM glucose, 25 mM HEPES, 25 mM sodium bicarbonate, heat-inactivated 10% fetal bovine serum (FBS; Peak Serum, Inc., Wellington, CO, USA), pen-strep cocktail (100 IU penicillin, 100 μg/mL streptomycin; Corning, Inc.), and 50 μM β-mercaptoethanol (MilliporeSigma, Burlington, MA, USA). The number of viable cells was counted by trypan blue exclusion, and 1 × 10^6^ viable cells per well were plated in 96-well round-bottom plates. The cells were cultured in the supplemented RPMI-1640 medium alone or with BLG at the concentration indicated (0, 1.0, or 10 mg/mL) for 72 h at 37°C with 5% CO_2_. The responsiveness of the cells to an immunological stimulus was validated using concanavalin A (ConA; data not shown).

### Immunophenotyping Using Flow Cytometry

The splenocytes culture plates were centrifuged at 400 × *g* for 5 min at 4°C, and the media were removed from the wells and stored at −80°C. The remaining cells were washed twice with PBS and incubated with Ghost Dye™ Violet 510 viability dye (1:1,000 in PBS; Tonbo Biosciences, San Diego, CA, USA) for 30 min on ice in the dark. After washing in PBS and blocking with anti-CD16/32 antibody (Biolegend, Cat# 101302) at a 1:50 dilution in PBS with 2% FBS, the cells were incubated in one of 3 designated antibody cocktails, each containing a panel of specific primary antibodies for immunophenotyping (Table 1). The cells were stained for 1 h at 4°C in the dark, washed in PBS with 2% FBS, and fixed for 1 h at 4°C in Fixation Buffer (Biolegend). For the antibody panel 3, which included an intracellular target (CD206), the fixed cells were washed in Perm/Wash Buffer (BioLegend), blocked with 3% rat serum in Perm/Wash Buffer for 10 min at 4°C in the dark, and incubated with anti-CD206 antibody diluted in Perm/Wash Buffer for 1 h at 4°C in the dark. Labeled cells were washed and resuspended in PBS containing 2% FBS and 1 mM EDTA and analyzed using a BD FACSymphony A3 flow cytometer (BD Biosciences, San Jose, CA, USA) at the North Dakota Flow Cytometry and Cell Sorting Core Facility. Unstained, single-stained, and fluorescence-minus-one (FMO) controls for every fluorochrome were used to set gating parameters to exclude dead cells, determine specificity, and establish the compensation panel to avoid spectral overlaps. At least 140,000 total cells were acquired.

**Table 1:** Antibodies for flow cytometric immunophenotyping.

**Target**	**Fluorophore**	**Dilution**	**Clone**	**Company**	**Catalog #**
B220	PE	2.5/100	RA3-6B2	BioLegend	103207
CD3	VF450	1/50	17A2	Tonbo Biosciences	75-0032-U100
CD4	BV605	1.25/100	RM4-5	BioLegend	100548
CD8	APC	1.25/100	53-6.7	BioLegend	100712
CD11b	APC	1.25/100	M1/70	BioLegend	101212
CD11c	BV711	0.25/100	N418	BioLegend	117349
CD14	BV605	1.25/100	RA3-6B2	BioLegend	123335
CD19	BV785	5/100	6D5	BioLegend	115543
CD25	PE	1/100	PC61	BioLegend	102008
CD45	PerCPCy5-5	1.25/100	30-F11	BioLegend	103132
CD49b	PE-Cy7	2.5/100	DX5	BioLegend	108921
CD80	PECY7	2.5/100	16-10A1	BioLegend	104733
CD86	FITC	2/100	GL-1	BioLegend	105005
CD206	PE	2.5/100	C068C2	BioLegend	141705
F4/80	BV785	2.5/100	BM8	BioLegend	123141
HLA-DR	FITC	1/20	L243	BioLegend	307604
HLA-DQ	FITC	1/20	HLADQ1	BioLegend	318104
IgM	APC	5/100	RMM-1	BioLegend	406509
Ly6G	PE-Cy7	1.25/100	1A8	BioLegend	127617
Ly6C	BV421	1/20	HK1.4	BioLegend	128031
TCRβ	FITC	2/100	H57-597	BioLegend	109205

The results were further analyzed using FlowJo v10.8.1 software (BD Life Sciences). For each panel, individual .fcs files from all samples were run on the same workspace. Using built-in FloJo plugins, .fcs files were inspected and cleaned for any anomalous signals and spurious events that might confound downstream data analysis. After gating on singlets from good events, the number of events from each sample was downsampled to the fewest number of live CD45^+^ events in each antibody panel. The minimum number of live/CD45^+^ for downsampling was determined based on the sample with the fewest numbers of CD45^+^ events. Downsampled CD45^+^ events from all samples were then selected for concatenation to define immune subpopulations (Supplementary Figure 1). The t-distributed stochastic neighbor embedding (t-SNE) was performed on the concatenated file using the default parameters in FloJo software (Supplementary Figure 2). To identify immune subsets and changes within genotypes and stimulation conditions, heatmaps with hierarchical clustering were generated with the heatmap tool, Morpheus (Supplementary Figure 3; software.broadinstitute.org/morpheus/), using fold change values and applying 1-Pearson correlation metric with average linkage in both columns and rows.

### Statistical Analysis

All statistical analyses were performed using GraphPad Prism v9.0 software (GraphPad Software, Inc., La Jolla, CA, USA), with males and females independently analyzed. *In vivo* intra-HLA strain differences were compared using multiple Mann-Whitney tests or multiple uncorrected *t*-tests as indicated in the figure legends. *In vivo* inter-HLA strain differences were compared using two-way ANOVA with Fisher's least significant difference (LSD) test. *In vitro* splenocyte experiments comparing unstimulated and stimulated splenocytes used a one-way ANOVA with a Dunnett's *post-hoc* test. The positive percentage of respective markers within each sample was first calculated, and the frequency of BLG-specific cells is presented as fold change by dividing the frequency of respective markers in the unstimulated condition. The ROUT method (Q = 1%) was used to identify outliers in a group when appropriate, and the values were removed from the final results. A *p* < 0.05 (*p* < 0.05) was considered statistically significant.

## Results

### HLA-II Allele and Sex Influenced Immediate Symptomatic Response to the BLG-Challenge

**Figure 2:**
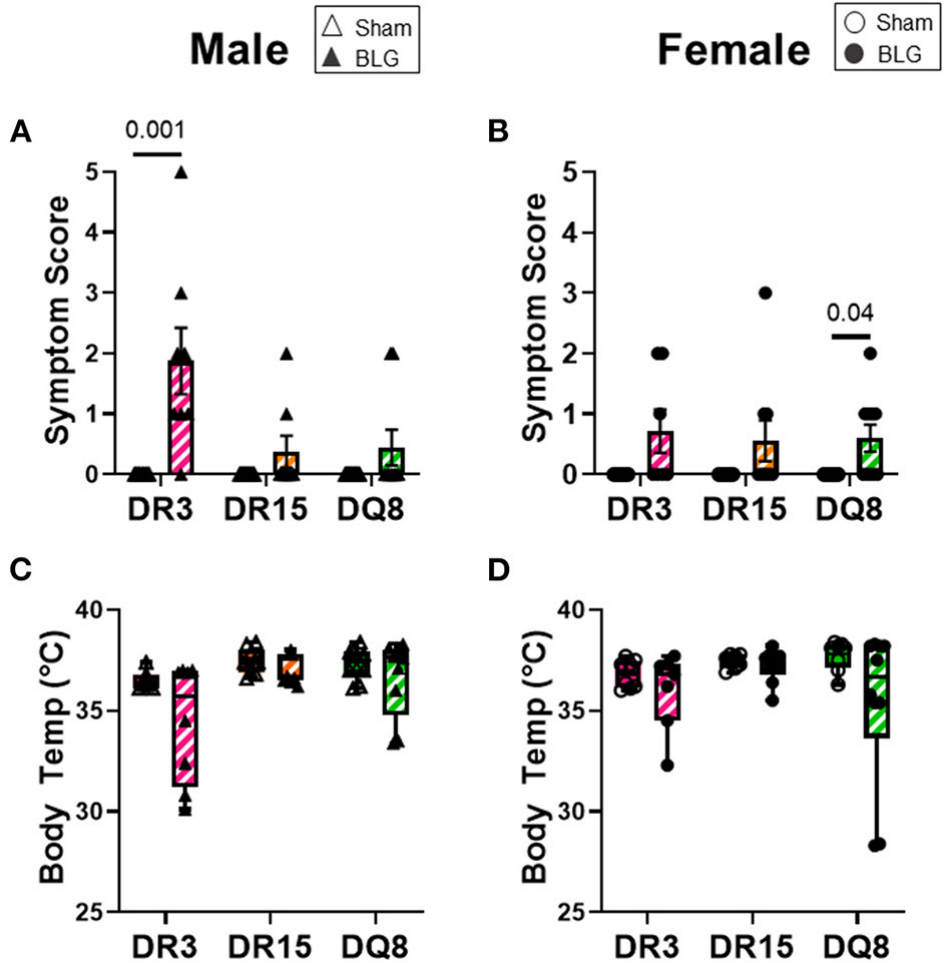
Acute physical symptoms upon allergen challenge. Symptomatic responses of sham and BLG-sensitized transgenic mice were observed 30 min after the oral BLG challenge based on a scale by Li et al. (36) **(A,B)**. Symptoms were scored as follows; 0: no observable symptoms; 1: scratching of head and ears; 2: edema, pilar erecti, and/or reduced activity; 3: labored respiration and cyanosis; 4: no activity or convulsions; 5: death. Body temperature was also measured at 30 min after the allergen challenge to observe the hypothermic response **(C,D)**. Sham mice (solid bars with open symbols); BLG mice (striped bars with closed symbols); HLA strain: DR3 (pink); DR15 (orange); DQ8 (green). Bars indicate group average values ± SEM **(A,B)**, while the box and whisker plots indicated the group average values and the maximum and minimum values **(C,D)** (*n* = 6–10 per group). Statistical significance (*p* < 0.05) was determined by multiple uncorrected Mann-Whitney tests. The numbers between lines indicate *p*-values.

As we have previously demonstrated with C57BL/6J and BALB/c mouse strains (18, 37), the growth of mice was unaffected by the BLG sensitization procedure in sham and BLG mice expressing HLA-II (Figures 1B,C). However, when challenged with a large dose of the allergen, the physical reactions of BLG-sensitized mice ranged widely by HLA-II strain and sex. On a symptom-based scale (36), BLG-sensitized DR3 males scored the highest by exhibiting the most severe reactions (BLG DR3: 1.9 ± 0.5; *p* = 0.001), with one mouse dying after the allergen challenge (Figure 2A). In contrast, BLG-sensitized DR15 and DQ8 males were largely asymptomatic, with their scores not significantly above their sham counterparts (Figure 2A; BLG DR15: 0.4 ± 0.3; BLG DQ8 0.4 ± 0.3). For all HLA-II strains, BLG-sensitized females showed only mild or no physical response after the allergen challenge (Figure 2B). However, the difference in the scores between sham and BLG-sensitized mice was significant for DQ8 females (BLG DQ8: 0.6 ± 0.2; *p* = 0.04) and near statistical significance for DR3 females (BLG DR3: 0.7 ± 0.4; *p* = 0.07). In addition to the symptom scoring, the hypothermic response was recorded 30 min post-challenge as an additional indicator of the immediate hypersensitivity response. While not statistically significant, BLG-sensitized DR3 males showed a clear trend for hypothermia (sham DR3: 36.5 ± 0.2°C; BLG DR3: 34.5 ± 1.0°C; *p* = 0.7) (Figure 2C). This decrease was also observed with BLG-sensitized DR3 females (sham DR3: 36.8 ± 0.2°C; BLG DR3: 36 ± 0.7°C; *p* = 0.6) and DQ8 females (sham DQ8: 37.7 ± 0.2°C; BLG DQ8: 35.4 ± 1.2°C; *p* = 0.2) (Figure 2D). These results suggested that the severity of the immediate physical reactions to the BLG challenge was both HLA-II and sex-dependent.

### BLG Sensitization Affected Mobility, Anxiety-Like Behaviors, and Spatial Memory of HLA-DR15 Mice in a Sex-Dependent Manner

**Figure 3:**
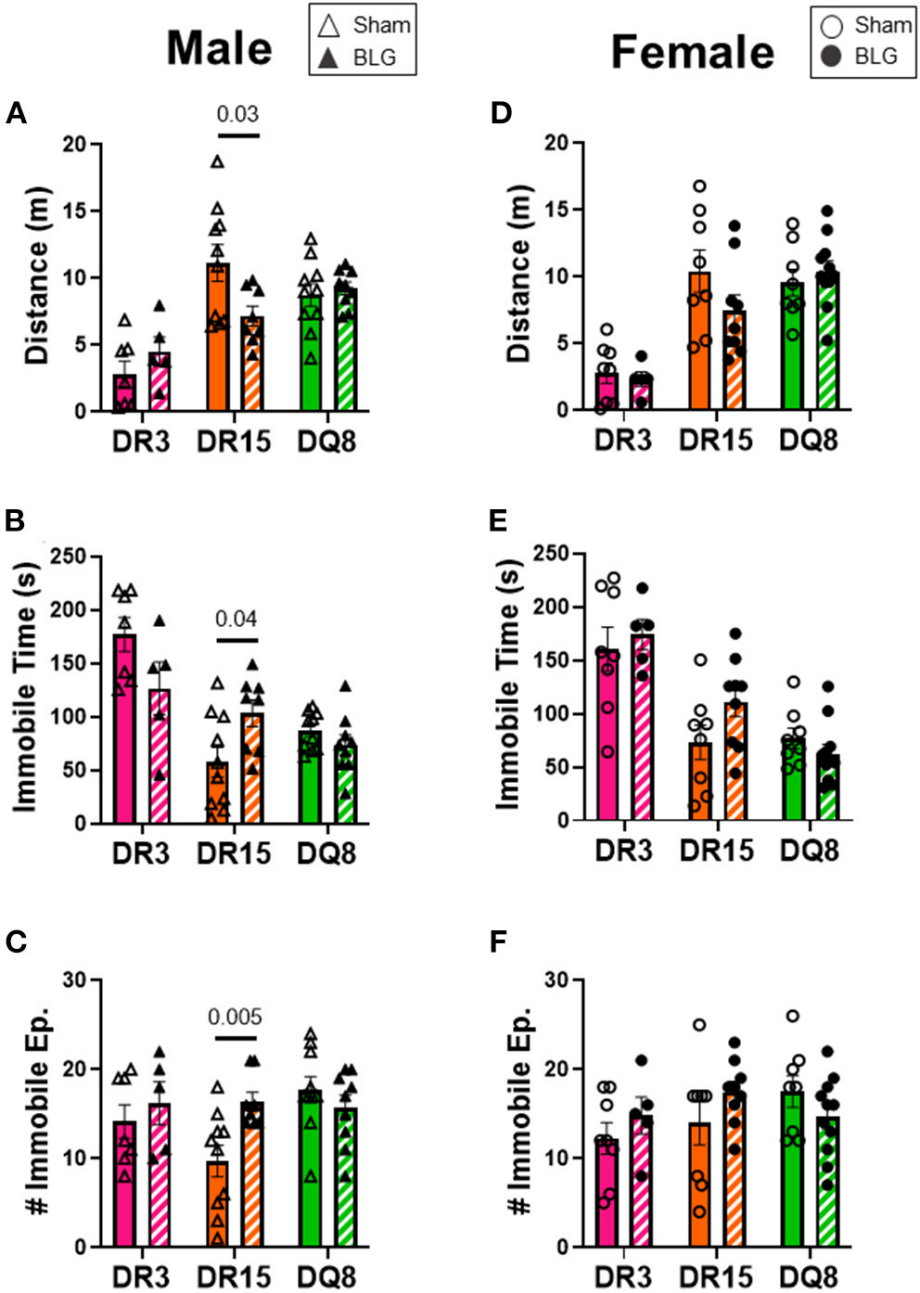
Activity monitoring with the open-field test (OFT). One day after the BLG challenge, sham and BLG-sensitized mouse behavior was observed with the OFT. The first 4 min of the test were analyzed to determine differences between sham and BLG treatment groups. Measurements included the total distance traveled **(A,D)**, immobile time **(B,E)**, and the number of immobile episodes **(C,F)** from males **(A–C)** and females **(D–F)**. Sham mice (solid bars with open symbols); BLG mice (striped bars with closed symbols); HLA strain: DR3 (pink); DR15 (orange); DQ8 (green). Bars indicate group average values ± SEM (*n* = 5–10 per group). Statistical significance (*p* < 0.05) was determined by multiple uncorrected Mann-Whitney tests. The numbers between lines indicate *p*-values.

**Figure 4:**
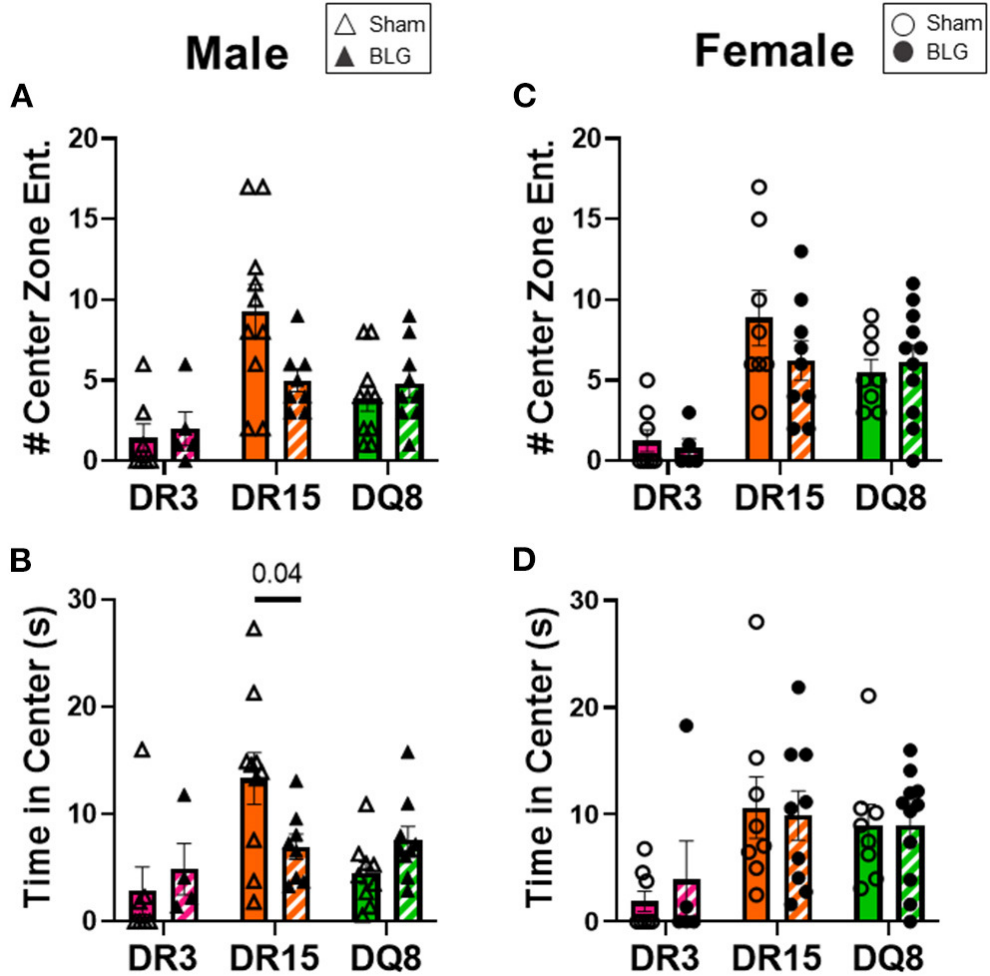
Anxiety-like behavior assessment with the open-field test (OFT). A center zone was defined in the OFT enclosure to assess anxiety-like behaviors in mice. The number of entries into the center zone **(A,C)** and the time spent in the zone **(B,C)** during the first 4 min of the test was measured from male **(A,B)** and female mice **(C,D)** in each experimental group. Avoidance of the center zone was considered anxiety-like behavior. Sham mice (solid bars with open symbols); BLG mice (striped bars with closed symbols); HLA strain: DR3 (pink); DR15 (orange); DQ8 (green). Bars indicate group average values ± SEM (*n* = 5–10 per group). Statistical significance (*p* < 0.05) was determined by multiple uncorrected Mann-Whitney tests. The numbers between lines indicate *p*-values.

**Figure 5:**
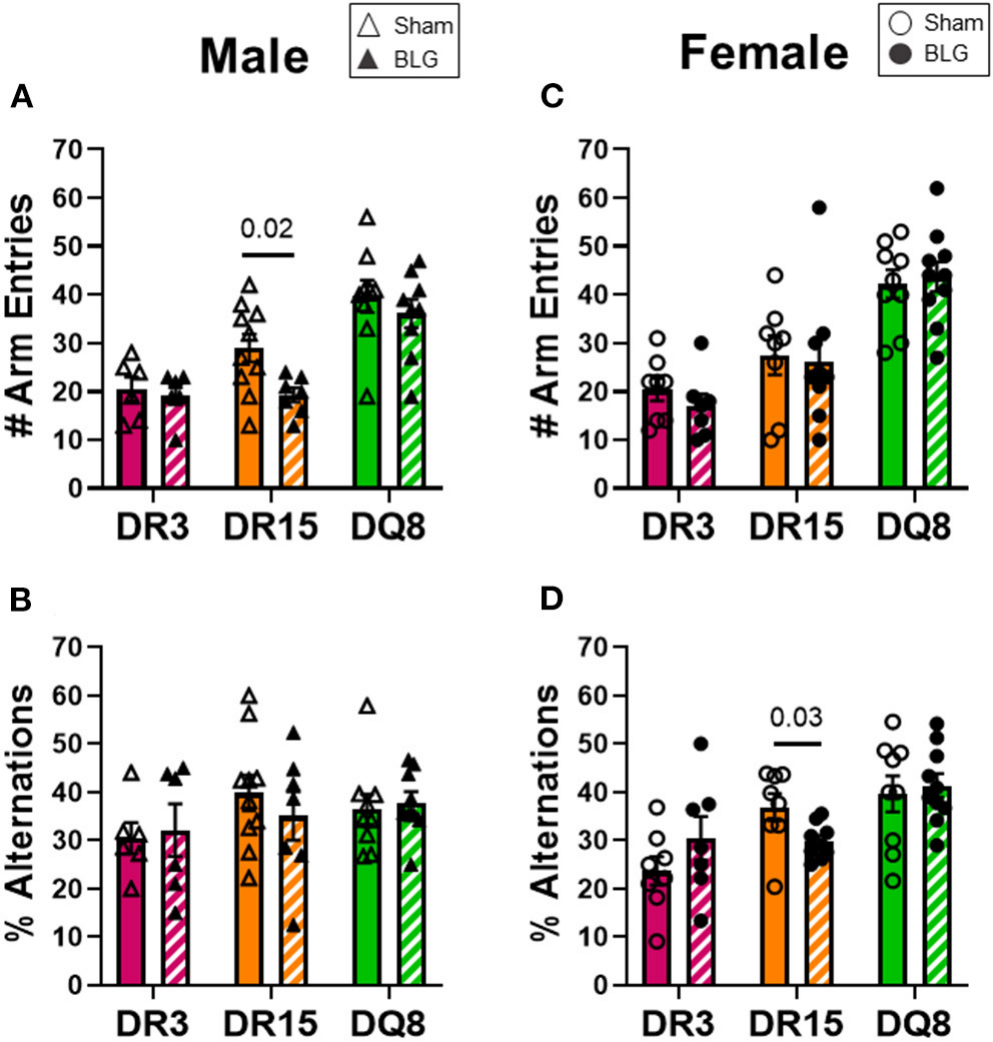
Spatial memory evaluation with the cross maze test (CMT). Mice were individually placed in a 4-arm maze and allowed to explore for 12 min. The number and sequence of entries to each arm were manually recorded by a blinded observer **(A,C)**, and the percent alternations for male and female mice of each experimental group was calculated **(B,D)**. A greater percentage of alternation indicated better spatial memory. Sham mice (solid bars with open symbols); BLG mice (striped bars with closed symbols); HLA strain: DR3 (pink); DR15 (orange); DQ8 (green). Bars indicate group average values ± SEM (*n* = 6–10 per group). Statistical significance (*p* < 0.05) was determined by multiple uncorrected Mann-Whitney tests. One sensitized male DR15 was found to be an outlier by ROUT and removed from the final results. The numbers between lines indicate *p*-values.

One day after the BLG-challenge, the general mobility of mice was assessed with the OFT, including the total distance traveled, time immobile, and the number of immobile episodes. Furthermore, anxiety-like behavior was also investigated by the avoidance and the time spent in the center zone of the OFT arena. We have previously reported that mice become acclimated to the OFT apparatus over the 10 min testing period (18). Thus, after quantifying their activities at each 1-min interval (not shown), the first 4 min of the testing period prior to the apparent acclimation between the treatment groups was analyzed. Of the BLG-sensitized male HLA-II mice, only the DR15 mice showed significant behavioral changes. Upon assessing the overall locomotion with the OFT (Figures 3A–F), the sensitized DR15 males traveled significantly less distance (Figure 3A; sham DR15: 11.1 ± 1.4 m; BLG DR15: 7.2 ± 0.7 m; *p* = 0.03) and spent nearly twice as long immobile (Figure 3B; sham DR15: 57.7 ± 14.0 s; BLG DR15: 103.5 ± 0.7 m; *p* = 0.04) with a greater number of immobile episodes than sham mice (Figure 3C; sham DR15: 10 ± 2; BLG DR15: 16 ± 1; *p* = 0.005). In addition, the sensitized DR15 females showed a trend for lower mobility than their sham counterparts (Figures 3D–F). When anxiety-like behavior was evaluated with the OFT (Figure 4), male DR15 mice showed a tendency toward entering the center zone less frequently (Figure 4A; sham DR15: 9 ± 2; BLG DR15: 5 ± 1; *p* = 0.08) and spent a shorter amount of time in the center than sham mice (Figure 4B; sham DR15: 13.3 ± 2.4 s; BLG DR15: 7.0 ± 1.2 s; *p* = 0.04), although no significant differences were observed in the female groups (Figures 4C,D). When spatial memory was evaluated by the CMT (Figures 5A–D), BLG-sensitized male DR15 mice exhibited decreased mobility as in the OFT, with fewer numbers of arm entries than the strain-matched sham mice (Figure 5A; sham DR15: 29 ± 3; BLG DR15: 19 ± 1; *p* = 0.02). However, the percent alternations by these mice were comparable between the treatment groups (Figure 5B), suggesting that their sensitization status did not influence their spatial memory after the allergen challenge. In contrast, while no differences in the number of arm entries were observed between the strain-matched female groups (Figure 5C), sensitized DR15 female mice showed significantly fewer successful alterations (Figure 5D; sham DR15: 36.9 ± 2.8%; BLG DR15: 29.8 ± 1.2%; *p* = 0.03) while their total number of arm entries was similar to the sham mice. Inter-HLA strain comparisons of the sensitized mice are shown in Supplementary Figures 4–6. These results indicated that the behavioral manifestations of CMA were observed only in BLG-sensitized DR15 mice when mice were challenged acutely, and changes in their behavioral phenotypes were sex-specific.

### Immunoglobulin Production of BLG-Sensitized Mice Was Distinct Among the Three HLA-II Strains

**Figure 6:**
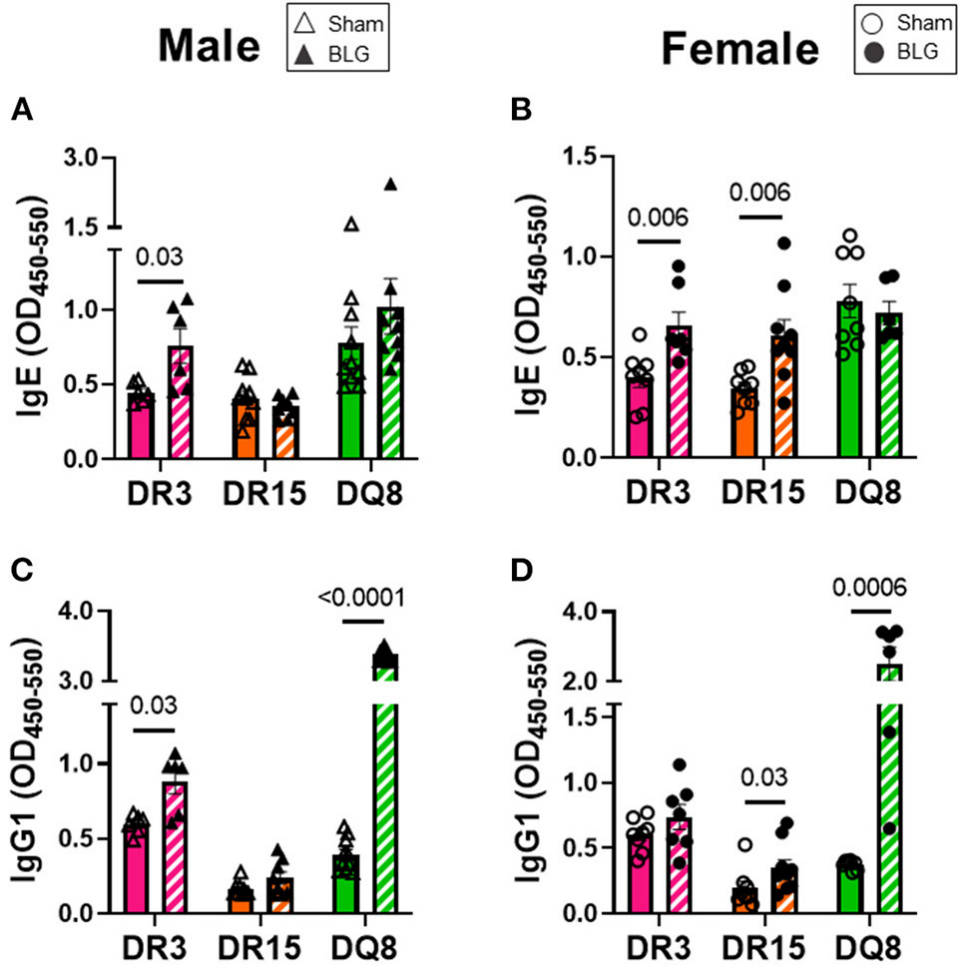
Allergen-specific IgE and IgG1 quantification. The levels of BLG-specific IgE **(A,B)** and IgG1 **(C,D)** in the sera isolated from terminal blood samples were determined by ELISAs. Sham mice (solid bars with open symbols); BLG mice (striped bars with closed symbols); HLA strain: DR3 (pink); DR15 (orange); DQ8 (green). Bars indicate group average values ± SEM (*n* = 6–10 per group). Statistical significance (*p* < 0.05) was determined by multiple uncorrected Mann-Whitney tests. The numbers between lines indicate *p*-values.

Circulating levels of BLG-specific IgE and IgG1 from terminal blood samples were quantified to assess hypersensitivity immune responses by the sham and sensitized mice. Allergen-specific IgE was significantly elevated by 2-fold in the sensitized DR3 male mice (sham DR3: 0.4 ± 0.03; BLG DR3: 0.8 ± 0.1; *p* = 0.03) but not in DR15 and DQ8 male mice (Figure 6A). However, when the serum samples from female mice were assayed, the levels of BLG-specific IgE were 1.7-fold greater in sensitized DR3 mice (sham DR3: 0.4 ± 0.05; BLG DR3: 0.7 ± 0.07; *p* = 0.006) and 2-fold in DR15 (sham DR15: 0.3 ± 0.03; BLG DR15: 0.6 ± 0.08; *p* = 0.006) compared to the strain- and sex-matched sham mice (Figure 6B). The allergen-specific IgE levels in sensitized DQ8 mice were comparable to the sham counterparts as observed in males (Figure 6B). On the other hand, BLG-specific IgG1 levels showed small but significant elevation in sensitized male DR3 mice compared to sham (Figure 6C; DR3 sham 0.6 ± 0.03; DR3 BLG 0.9 ± 0.08; *p* = 0.03) and in sensitized female DR15 mice (Figure 6D; DR15 sham 0.2 ± 0.05; DR15 BLG 0.4 ± 0.06; *p* = 0.03). Interestingly, the allergen-specific IgG1 levels were robustly elevated in both male and female DQ8 sensitized mice, with approximately 8.5- and 6-times greater levels of IgG1, respectively, than their sham mice (Figures 6C,D; male sham DQ8: 0.4 ± 0.04; male BLG DQ8: 3.4 ± 0.03; *p* < 0.0001; female sham DQ8: 0.4 ± 0.01; female BLG DQ8: 2.5 ± 0.5; *p* = 0.0006). Inter-HLA strain comparisons are shown in Supplementary Figure 7. Together, these results indicated that the humoral response to the BLG allergen was affected both by the HLA-II allele expressed and sex.

### Serum MCPT-1 Levels Varied by HLA-II Strain and Sex

**Figure 7:**
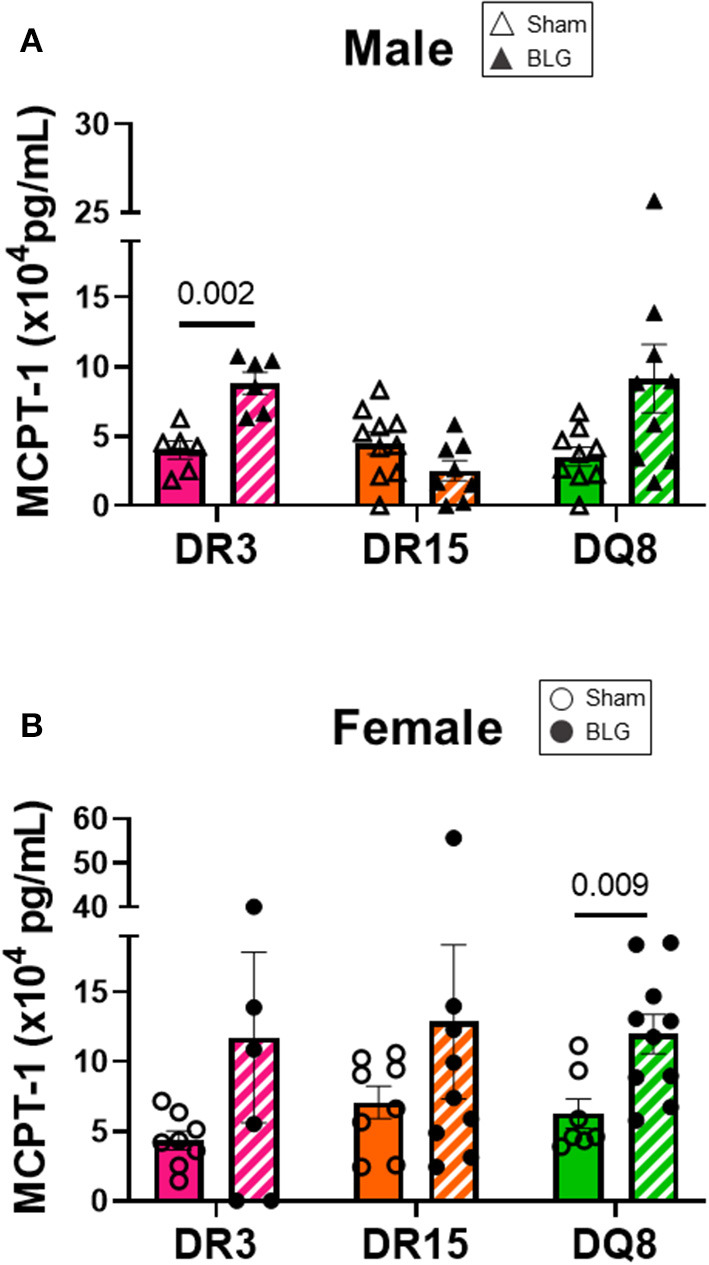
Serum mast cell protease-1 (MCPT-1) levels. Terminal blood samples from male **(A)** and female **(B)** mice were used to detect MCPT-1 by ELISA. Sham mice (solid bars with open symbols); BLG mice (striped bars with closed symbols); HLA strain: DR3 (pink); DR15 (orange); DQ8 (green). Bars indicate group average values ± SEM (*n* = 6–10 per group). Statistical significance (*p* < 0.05) was determined by multiple uncorrected Mann-Whitney tests. The numbers between lines indicate *p*-values.

Mast cells are the effector cells of the immediate allergic response and release their granular contents, such as histamine and proteases, upon allergen binding to their cell-surface IgE-FcεRI complex (41). To compare the differences in mast cell activation in the three HLA-II transgenic strains, we measured MCPT-1 from the serum samples of sham and BLG-sensitized mice. MCPT-1 was significantly elevated in sensitized DR3 male mice by 2-fold (Figure 7A; sham DR3: 4.0 ± 0.7 pg/mL; BLG DR3: 8.8 ± 0.8 pg/mL; *p* = 0.002). Sensitized female mice exhibited notable variabilities in the MCPT-1 levels within each strain. In particular, one of the sensitized DR3 and DR15 mice showed a much greater level of MCPT-1 than others in the respective group (Figure 7B). However, only the MCPT-1 levels in sensitized HLA-DQ8 mice were significantly different from the sham mice (Figure 7B; sham DQ8: 6.3 ± 1.1 pg/mL; BLG DQ8: 12.8 ± 5.5 pg/mL; *p* = 0.009). Inter-HLA strain comparisons are shown in Supplementary Figure 8. These observations suggested that mast cells were differentially activated among the three strains of HLA-II mice, resulting in the varying levels of mast cell-derived proteases in their circulation at the time of euthanasia.

### The Composition of Circulating Leukocytes Was Differentially Altered Among the HLA-II Transgenic Strains With BLG Sensitization

**Figure 8:**
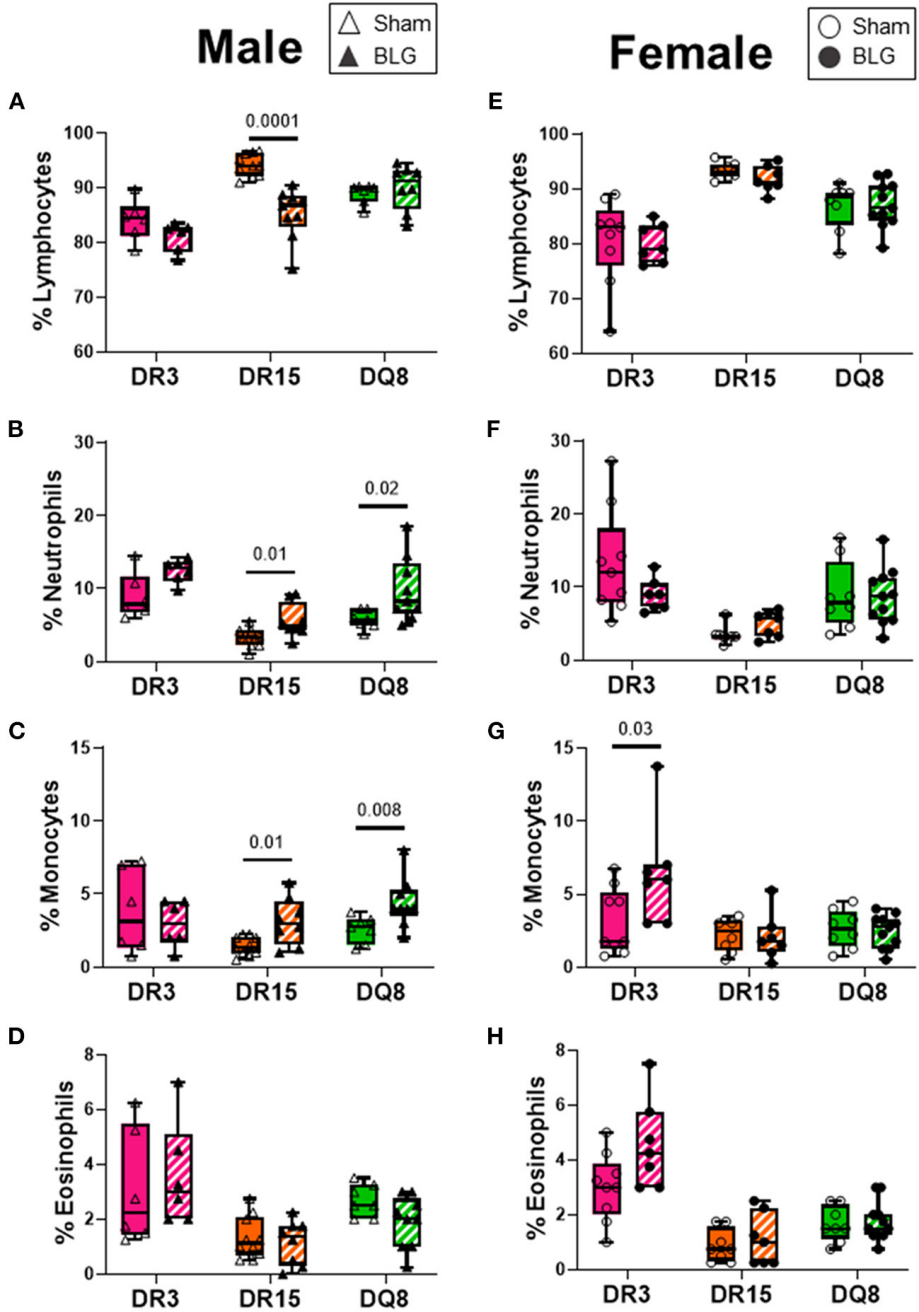
Blood smear leukocyte differentials. EDTA-anticoagulated terminal whole blood was used to make a thin smear and stained with Wright-Giemsa stain to differentiate white blood cell populations. Two blinded observers counted lymphocytes, neutrophils, monocytes, and eosinophils, and the relative percentages of each cell type were calculated for male **(A–D)** and female **(E–H)** mice. Sham mice (solid bars with open symbols); BLG mice (striped bars with closed symbols); HLA strain: DR3 (pink); DR15 (orange); DQ8 (green). The box and whisker plots indicate group average values and the minimum and maximum values (*n* = 6–10 per group). Statistical significance (*p* < 0.05) was determined by multiple uncorrected Mann-Whitney tests. The numbers between two bars indicate the *p*-values.

Blood smears were prepared at the time of sacrifice to investigate changes in circulating leukocyte composition (Figures 8A–H). HLA-DR15 mice had the most dynamic change among the BLG-sensitized male mice, with a 3% decrease in circulating lymphocytes (Figure 8A; sham DR15: 84.1 ± 1.5%; BLG DR15: 81.0 ± 1.1%; *p* = 0.0001). In addition, a 2% increase in neutrophils (Figure 8B; sham DR15: 3.3 ± 0.4%; BLG DR15: 5.6 ± 0.8%; *p* = 0.01) and approximately 1.5% more monocytes were observed in these mice than their sham counterparts (Figure 8C; sham DR15: 1.5 ± 0.2%; BLG DR15: 3.1 ± 0.6%; *p* = 0.01). BLG-sensitized male DQ8 mice had 4% greater number of neutrophils (Figure 8B; sham DQ8: 5.8 ± 0.5%; BLG DQ8: 9.9 ± 1.5%; *p* = 0.02) and an almost 2% increase in monocytes (Figure 8C; sham DQ8: 2.6 ± 0.3%; BLG DQ8: 4.4 ± 0.6%; *p* = 0.008). Among the BLG-sensitized female groups, only HLA-DR3 mice showed significant changes in their blood leukocytes, with a 3% increase in circulating monocytes (Figure 8G; sham DR3: 3.1 ± 0.7%; BLG DR3: 6.4 ± 1.4%; *p* = 0.03). Although the immediate reaction to the challenge had occurred over 2 days prior to sacrifice, small but significant differences in blood leukocyte composition were detectable, particularly in the male sensitized transgenic mice. These findings suggest that these persisting changes occurred during the sensitization or were triggered by the allergen challenge.

### BLG-Challenge Increased Intestinal Inflammation in HLA-II and Sex-Specific Manner

**Figure 9:**
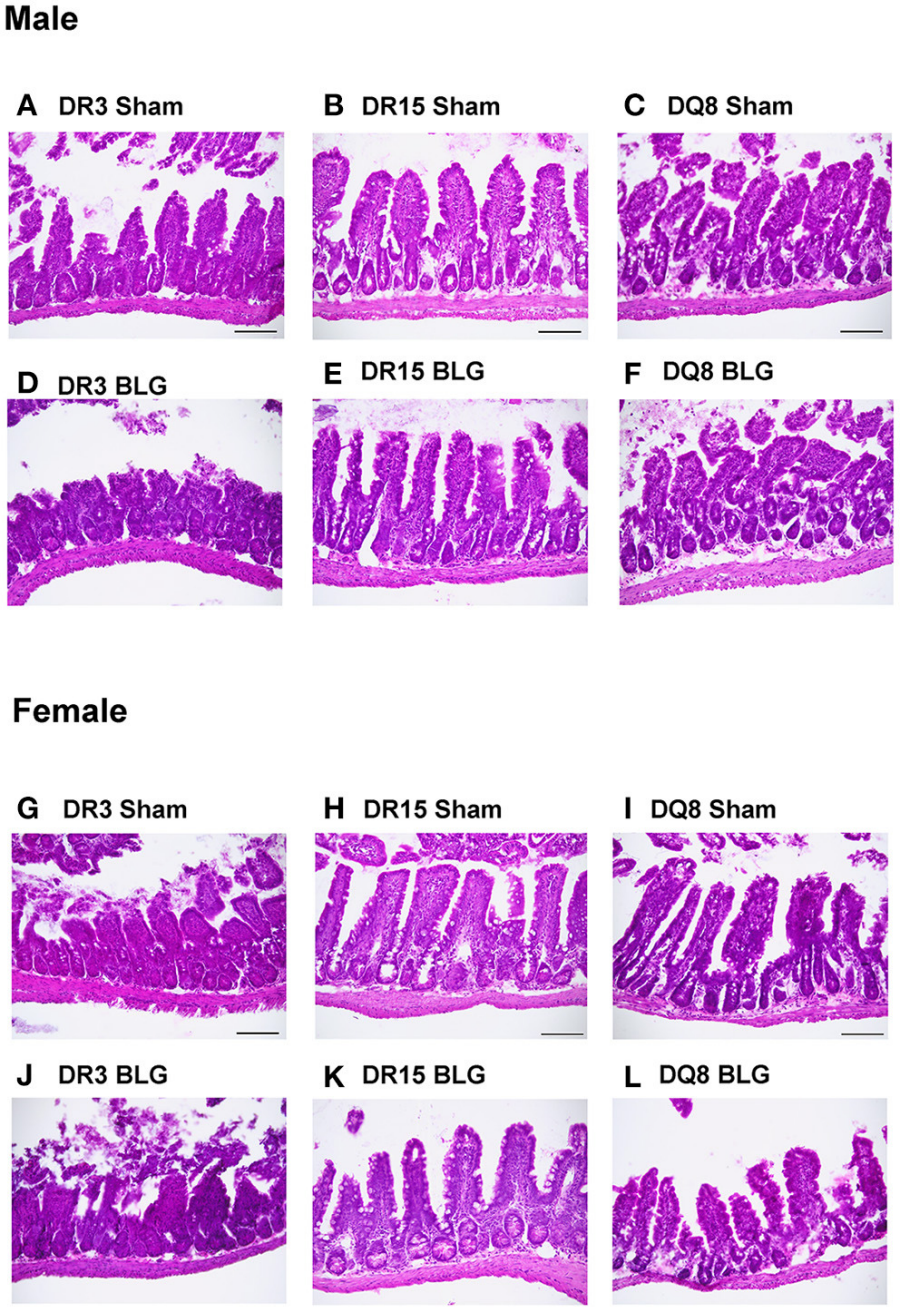
Hematoxylin and eosin (H&E) stained ileum. Ileum sections from the transgenic mice were stained with H&E to visualize the morphology of the gastrointestinal tract. Representative photomicrographs for male sham **(A–C)**, male BLG **(D–F)**, female sham **(G–I)**, and female BLG **(J–L)** mice from each of the HLA-II transgenic strains were taken using a 20x objective (scale bar = 100 μm).

**Figure 10:**
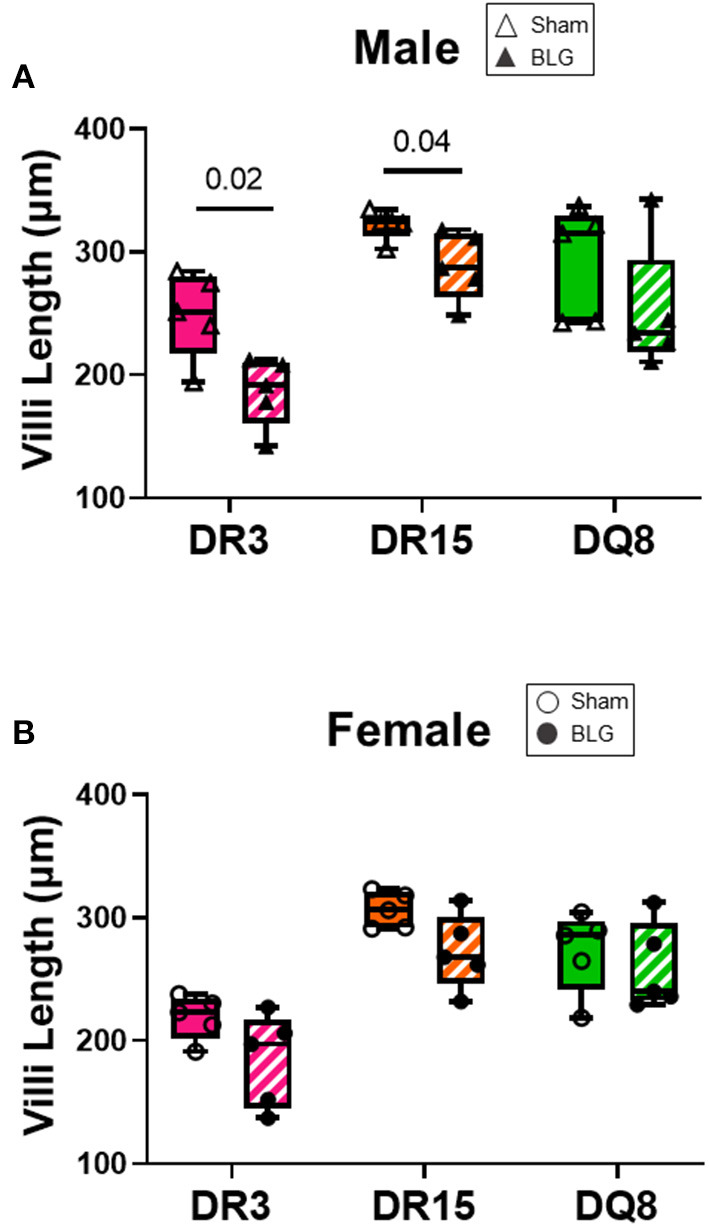
Ileum villi length. H&E-stained ileal sections from male **(A)** and female **(B)** mice were scanned, and the whole-slide images were used to measure the villi length using NDP.view2 software The average villi lengths were calculated for each experimental group. BLG mice (striped bars with closed symbols); HLA strain: DR3 (pink); DR15 (orange); DQ8 (green). The box and whisker plots indicate group average values and the minimum and maximum values (*n* = 5 per group). Statistical significance (*p* < 0.05) was determined by multiple uncorrected *t*-tests. The numbers between lines indicate *p*-values.

Ileum sections were stained with H&E to determine intestinal inflammatory changes due to the BLG sensitization and challenge among the transgenic mouse strains. While inflammation was evident in all the ileums of BLG-sensitized male (Figures 9A–F) and female mice (Figures 9G–L), the extent of the structural changes depended on HLA-II expression and sex. BLG-sensitized DR3 males were the most severely affected with highly disorganized mucosal/submucosal architecture (Figures 9A,D) and significant blunting of the villi (Figure 10A; sham DR3: 249 ± 16 μm; BLG DR3: 187 ± 13 μm; *p* = 0.02). Sensitized DR15 males also showed a significant decrease in villi length (Figure 10A; sham DR15: 322 ± 5 μm; BLG DR15: 289 ± 12 μm; *p* = 0.04), though the changes in their overall mucosa and submucosa morphology were less extensive (Figures 9B,E). Disorganized villi and crypt structure were also noticeable in DQ8 male mice (Figures 9C,F), but the trend of decrease in their average villi length did not reach statistical significance (Figure 10A; sham DQ8: 292 ± 20 μm; BLG DQ8: 251 ± 23 μm). In female ileums, BLG-sensitized DR3 mice again had the most drastic structural changes in the mucosal and submucosal organization (Figures 9G,J), while overall morphology of the sensitized DR15 and DQ8 ileums were similar to their respective sham controls (Figures 9H,I,K,L). There was no significant shortening of the villi in any of the female HLA-II strains, though BLG-sensitized mice tended to have shorter villi than their sham counterparts (Figure 10B). While the BLG challenge increased inflammatory changes in sensitized mice of all the transgenic strains, the severity of the changes was greatly influenced by both the HLA-II strain and the sex of the sensitized mice.

### Direct BLG Stimulation of Immune Cells Produce HLA- and Sex-Specific Responses in a Concentration-Dependent Manner

**Figure 11:**
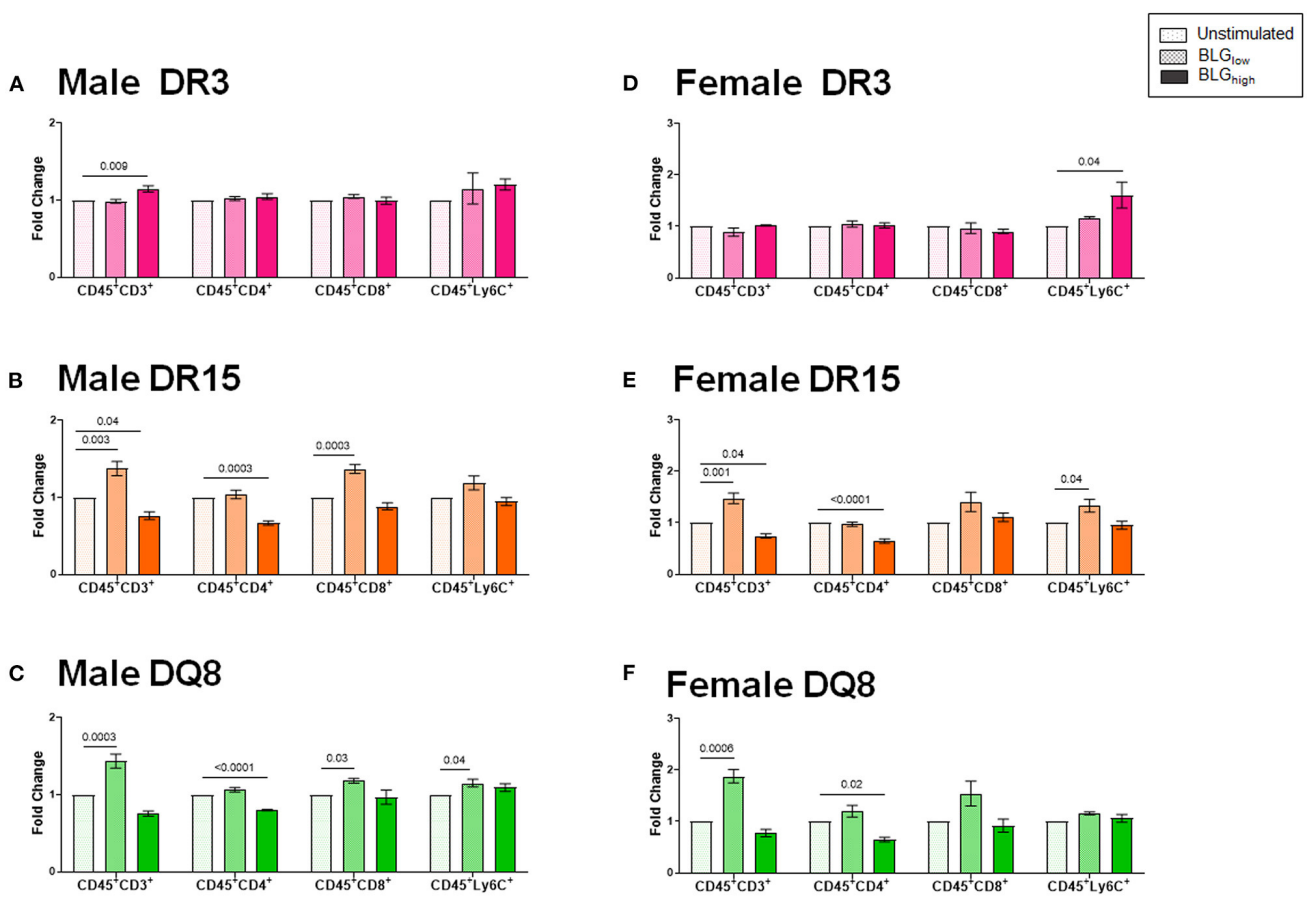
Flow cytometric quantitation of immunophenotype profiles identified as Cluster 1. Splenocyte preparations from naïve male **(A–C)** and female **(D–F)** transgenic mice were stimulated with BLG at 1 mg/mL (BLG_low_) or 10 mg/mL (BLG_high_) for 72 h. Cells were stained for flow cytometric immunophenotyping. One of the immune profile groups identified by Morpheus-based hierarchical clustering was designated as Cluster 1, and BLG-stimulated changes in the population were expressed as fold change from the sex- and strain-matched unstimulated cell groups. BLG concentration: Unstimulated (open dotted pars); BLG_low_ (checkered bars); BLG_high_ (solid bars); HLA strain: DR3 (pink); DR15 (orange); DQ8 (green). Bars indicate group average values ± SEM (*n* = 3–5 per group). Statistical significance (*p* < 0.05) was determined by one-way ANOVA with Dunnett's *post-hoc* test. The numbers between lines indicate *p*-values.

**Figure 12:**
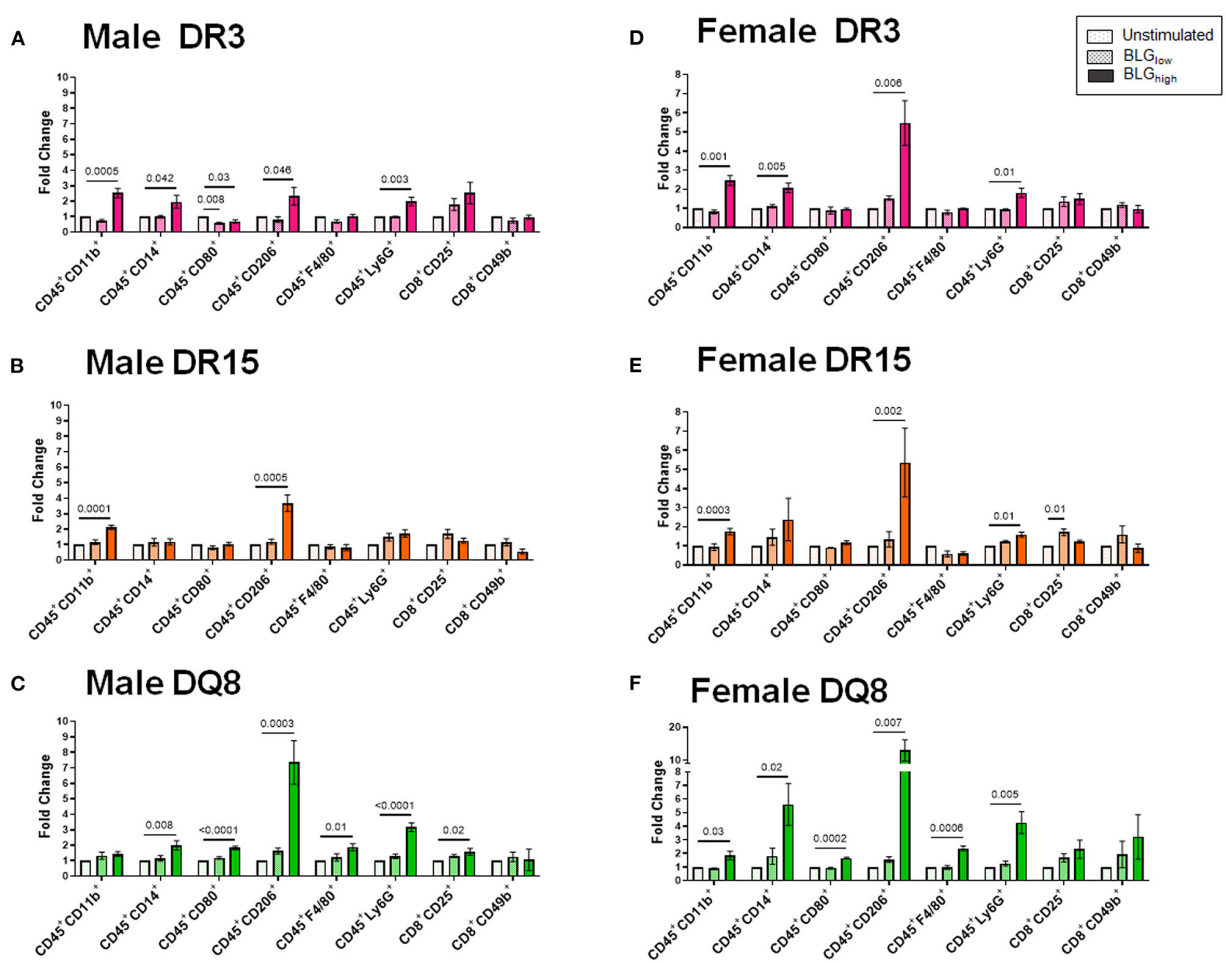
Flow cytometric quantitation of immunophenotype profiles identified as Cluster 2. Splenocyte preparations from naïve male **(A–C)** and female **(D–F)** transgenic mice were stimulated with BLG at 1 mg/mL (BLG_low_) or 10 mg/mL (BLG_high_) for 72 h. Cells were stained for flow cytometric immunophenotyping. One of the immune profile groups identified by Morpheus-based hierarchical clustering was designated as Cluster 2, and BLG-stimulated changes in the population were expressed as fold change from the sex- and strain-matched unstimulated cell groups. BLG concentration: Unstimulated (open dotted pars); BLG_low_ (checkered bars); BLG_high_ (solid bars); HLA strain: DR3 (pink); DR15 (orange); DQ8 (green). Bars indicate group average values ± SEM (*n* = 3–5 per group). Statistical significance (*p* < 0.05) was determined by one-way ANOVA with Dunnett's *post-hoc* test. The numbers between lines indicate *p*-values.

**Figure 13:**
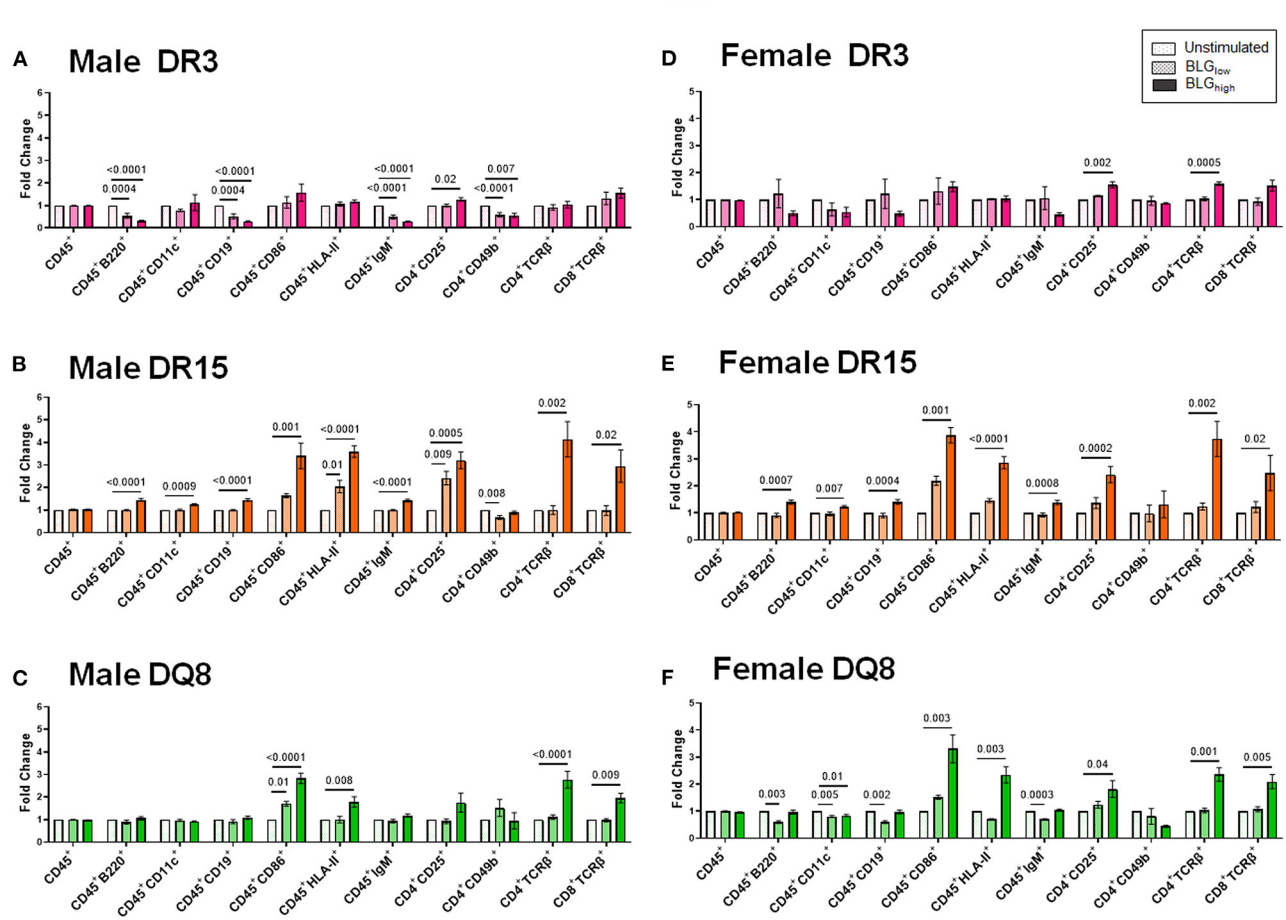
Flow cytometric quantitation of immunophenotype profiles identified as Cluster 3. Splenocyte preparations from naïve male **(A–C)** and female **(D–F)** transgenic mice were stimulated with BLG at 1 mg/mL (BLG_low_) or 10 mg/mL (BLG_high_) for 72 h. Cells were stained for flow cytometric immunophenotyping. One of the immune profile groups identified by Morpheus-based hierarchical clustering was designated as Cluster 3, and BLG-stimulated changes in the population were expressed as fold change from the sex- and strain-matched unstimulated cell groups. BLG concentration: Unstimulated (open dotted pars); BLG_low_ (checkered bars); BLG_high_ (solid bars); HLA strain: DR3 (pink); DR15 (orange); DQ8 (green). Bars indicate group average values ± SEM (*n* = 3–5 per group). Statistical significance (*p* < 0.05) was determined by one-way ANOVA with Dunnett's *post-hoc* test. The numbers between lines indicate *p*-values.

To better understand the influence of BLG on the immune responses of the transgenic mouse strains as an antigen, we directly stimulated splenocytes from naïve mice with either 1 mg/mL (BLG_low_) or 10 mg/mL (BLG_high_) of BLG *in vitro*. The phenotypic changes in BLG-stimulated cells were expressed as fold changes from the strain- and sex-matched unstimulated controls (Figures 11–**13**), and the samples were classified according to the similarities in their expression profiles of several markers using Morpheus. The algorithm-assisted hierarchical clustering grouped the phenotypic markers based on distinct concentration- and genotype-specific immune profiles and revealed three major profiles of immune subsets (Supplementary Figure 3).

The first cluster grouped phenotypic markers for T cells (CD3^+^, CD4^+^, CD8^+^) and myeloid cells (Ly6C^+^), such as dendritic cells, monocytes, and/or macrophages (Figures 11A–F). As anticipated, the splenocytes from the three HLA-II variant strains of male and female mice altered their immunophenotypes in the presence of the allergen. While BLG did not elicit notable changes in the T cell populations in the splenocytes from DR3, particularly from female cells (Figures 11A,D), the allergen altered the T-cell-associated profiles in DR15 and DQ8 of both sexes. The effects of BLG on the CD3^+^ population in DR15 and DQ8 were concentration-dependent, with the expansion of this population promoted by BLG_low_ and suppressed by BLG_high_. The Ly6C^+^ populations in the cells from these genotypes showed similar profile changes. However, female DR3 splenocytes were stimulated to expand this population at the higher concentration of BLG.

Cluster 2 largely grouped innate markers, including CD11b^+^, F4/80^+^, CD14^+^, CD206^+^, Ly6G^+^, CD80^+^, and the innate-to-adaptive bridging phenotype, CD8^+^CD49b^+^, and the effector phenotype, CD8^+^CD25^+^. Among this group, the most prominent finding was that BLG_high_ induced profound expansion of CD45^+^CD206^+^ cells in the stimulated splenocytes from all three genotypes of both sexes (Figure 12). In addition, concentration-dependent increases were observed with the CD45^+^CD11b^+^ cell populations in all but male DQ8 splenocyte preparations after stimulation with BLG_high_. In contrast, the expansion of leukocyte populations labeled with the neutrophil marker, Ly6G, increased with BLG_high_ in all samples except in splenocytes from male DR15. Furthermore, the cells from male and female DR15 failed to expand the CD14^+^ population, indicating that the allergen did not stimulate monocytes in this genotype even at the higher concentration of BLG. DQ8 was uniquely stimulated by BLG_high_, showing increased F4/80^+^ macrophage and CD80^+^ leukocytes populations, regardless of sex.

The phenotypes identified by the third cluster included CD45^+^, CD11c^+^, HLA-II^+^, CD4^+^CD49b^+^, CD4^+^CD25^+^, CD4^+^TCRβ^+^ (TCRβ: the variable beta chain of the T cell receptor), CD8^+^TCRβ^+^, CD86^+^, B220^+^, CD19^+^, and IgM^+^. Among this group, CD4^+^TCRβ^+^, CD8^+^TCRβ^+^, CD86^+^, and HLA-II^+^ populations were uniquely and significantly increased in the splenocytes from DR15 and DQ8, but not DR3, for both sexes (Figure 13). Additionally, the population of B cells expressing B220, CD19, and IgM were also upregulated by BLG_high_ in the splenocytes from male and female DR15 mice but not in any other samples. These results suggested that the expansion of B cell populations likely facilitated T cell activation in a manner specific to the HLA-II variant, particularly highlighting the classic HLA-II/TCR interactions and cognate B cell/CD4^+^ T cell interactions in DR15. Innate responses were more pronounced in DQ8.

Together our *in vitro* results indicated that BLG, especially at a higher concentration, stimulated innate and adaptive immune responses differentially among the three genotypes, suggesting that the HLA-II allele dictates the downstream cellular responses.

## Discussion

Food allergy is highly variable in both symptomatic presentation and severity, and many factors may contribute to the inconsistent manifestations among the allergic population (2, 42, 43). Previously, we observed that sensitizing C57BL/6J and Balb/cJ mice to the same milk allergen resulted in strain-specific physical symptoms, behavioral manifestations, immune response, and microbiome alterations (18). These experimental mouse strains have distinct genetic backgrounds and immune responses (17, 19, 20). Based on these findings, we sought to investigate further the role of immunogenetics in food allergy symptom manifestation more directly relevant to humans. Therefore, in this study, we utilized transgenic HLA-II mice and established humanized CMA mouse models to determine whether the variations in HLA-II alleles would influence the presentation of allergic symptoms, behavioral changes, and immune responses. We also characterized the sex- and variant-specific immune responses to BLG *in vitro*.

We first examined the role of HLA-II alleles on immediate physical responses to an allergen challenge. The acute reactions to the allergen challenge by BLG-sensitized DR3 male mice were most apparent (Figures 2A,C), which were corroborated by the significant increases in the allergen-specific IgE and MCPT-1 levels detected in the terminal blood of these animals (Figures 6, 7). However, the severity of immediate hypersensitivity responses did not always correlate with the levels of BLG-specific immunoglobulins or MCPT-1 in all transgenic strains or sexes. In particular, the relatively asymptomatic DR15 females had significantly greater IgE compared to their sham group (Figure 6B). In addition, although it did not reach statistical significance, the MCPT-1 levels in the majority of sensitized DQ8 male mice were higher than in their sham group (Figure 7A), despite the absence of their immediate reactions to the allergen (Figure 2A). The lack of severe anaphylaxis in these mice may potentially be explained by the elevation in their BLG-specific IgG1 levels. Although IgG subtypes have been shown to contribute to anaphylaxis in animals passively immunized and intravenously challenged mice (44–46), IgG has also been shown to play a protective role against IgE-mediated severe food allergy reactions, either by competing with IgE for the allergen or binding to FcγRIIb on granulocytes to hinder IgE binding to FcεRI (47, 48).

These results have shown significant variabilities in how sensitized individuals respond to the offending allergen. In addition, our observations, at least from examining the sera collected 2 days after the allergen challenge, suggested that the allergen-specific immunoglobulin and MCPT-1 levels in the blood may not necessarily indicate or predict the physical responses of sensitized individuals. The lack of clear correlation between the physical responses and the levels of these serum immune factors has been previously reported in allergen-sensitized wild-type strains after hours to days post-allergen challenge or *Was*^−/−^ transgenic mice on an allergen-containing diet (40, 49, 50). Further allele-specific examination of immunoglobulin production dynamics before, during, and after the allergic sensitization is required to fully elucidate their potential roles in symptom presentation and resolution.

In addition to their immediate physical symptoms, BLG-sensitized mice also showed HLA-II-dependent manifestation of behavioral changes. Interestingly, only sensitized DR15 mice showed changes in their behavior compared to their sex- and strain-matched sham mice, even though they were relatively asymptomatic immediately after the allergen challenge (Figure 2A). These behavior differences were also sex-specific, with sensitized DR15 males showing significantly decreased exploration and overall activities (Figures 3–5), while DR15 females had decreased spatial memory (Figure 5D). In our past studies using similar allergen sensitization and challenge paradigms, we have only detected significant behavioral changes in male mice (18, 37, 51, 52), largely related to anxiety- or depression-like behavior. While the brain pathophysiology of these transgenic mice was not examined in the present study, it is important to note that the HLA-DR15 haplotype is a risk factor for developing MS in humans (25–27). Driven by autoreactive immune cells that cause inflammation and demyelination of the central nervous system (53), various motor and mood changes have been reported in MS patients (54, 55). As food allergy is not only associated with various neuropsychiatric disorders (11, 12, 15, 16) but also is a chronic inflammatory condition, CMA may contribute to the susceptibility of individuals with HLA-DR15 to neuroinflammatory disorders, such as MS. Additional studies are warranted to determine the potential influence of specific HLA-II variants on neuroinflammation in food allergy and other inflammatory conditions. In particular, examining motor function, neuroinflammation, and demyelination in DR15 mice will determine whether the behavioral changes observed in this strain were the consequences of neuropathology influencing affective and/or motor deficits. Such a study will also raise the possibility that the DR15 allele may predispose individuals with CMA to manifestations of behavioral abnormalities.

The causal role of food allergy in behavioral changes has been suspected for decades (9–16), and the fear of accidental allergen exposure has been postulated to trigger anxiety and depression in allergic individuals who have previously experienced severe reactions (11, 56). However, earlier case reports described behavioral symptoms other than anxiety and depression, such as tics (13, 14) and hyperactivity (10–12). Furthermore, symptom resolution has also been reported in patients with mood and behavioral problems by elimination diets (57, 58), indicating that these patients were tolerant of the offending foods and did not suffer from severe reactions. In preclinical models of food allergy, we and others have shown that sensitization of otherwise healthy animals to food allergens resulted in behavioral abnormalities with heightened immune responses even though the sensitized mice did not exhibit anaphylaxis upon acute allergen challenge (18, 37, 51, 52). These observations in patients and animal models suggest that learned fear alone cannot explain the association between food allergy and behavioral symptoms. Nevertheless, the outcomes from clinical studies investigating the correlation of food allergy with mood and behavioral disorders have been inconsistent. The outcomes from our present study support the notion that the variabilities difficult to control with patient cohorts, such as genetic background, symptom types and severities, offending allergens, and diets, likely contribute to such inconsistency.

In addition to immediate responses and behavior, we also compared circulating leukocytes and intestinal pathologies of the three transgenic strains. We found that BLG-sensitized male mice, regardless of their HLA-II allele, showed more blood leukocyte changes than female mice (Figure 8). While these changes were only 2–3% above or below their sham counterparts, the normal circulating leukocyte population contains a relatively small percentage of the neutrophils, monocytes, and eosinophils compared to humans (59, 60). Thus, even the seemingly small changes could potentially impact pathophysiology in mice. Furthermore, because blood leukocytes tend to migrate rapidly to the site of tissue inflammation (61, 62), we might not have detected robust changes as we would, especially in circulating eosinophils or basophils, if the blood was sampled after the first few hours of BLG challenge. Unfortunately, it was difficult to collect peripheral blood from some of the sensitized mice shortly after the challenge due to their hypothermic response (Figures 2C,D).

However, in the ileum samples from sensitized mice, we observed that their mucosa/submucosa structures were clearly disorganized regardless of their strain (Figure 9). In particular, significant shortening of the villi was detected in the sensitized DR3 and DR15 males (Figure 10A). Similar observations have been reported after allergic sensitization and in other intestinal inflammatory conditions (63–65). Given that classic phagocytic leukocytes, such as neutrophils and monocytes, were increased in the circulation of sensitized males in our mouse model, these immune cell subtypes were likely recruited to the intestine to “clean up” after the allergic insult or promote further antigen presentation (66, 67). Nonetheless, sex- and HLA-II-specific changes were also detected in CMA-associated development of intestinal pathology.

Sex- and allele-dependent immunologic responses were also observed with our *in vitro* experiment using splenocyte preparations from the three transgenic strains. Immunophenotypes of leukocytes showed activation profiles that were variable across the groups after BLG stimulation (Figures 11–13). Given their mild response to the allergen challenge *in vivo*, it was intriguing to find that DR15 and DQ8 male splenocytes exhibited more robust T cell responses than their female counterparts or the DR3 strain by expanding the CD4^+^ and CD8^+^ T cell subsets upon BLG_high_ stimulation. Furthermore, these groups of cells also elevated the leukocyte populations expressing the costimulatory molecules, CD80 and CD86, upon BLG stimulation.

In addition to interactions between T cells and APCs, the magnitude of response to antigens/allergens relies on the activation of these costimulatory molecules expressed on APCs. These molecules serve as a secondary signal for T cell activation, conferring optimal adaptive immune responses (68). Our *in vitro* data strongly suggested that responses to BLG stimulation depended on HLA-II, which had presented the allergen. For example, in DQ8 mice, BLG_low_ was sufficient to trigger significant increases in the population expressing CD80, which, in tandem with CD86, can induce long-lasting effects. Likewise, in DR15 mice, BLG_low_ was able to induce the expansion of the populations expressing the markers associated with a classical antigen presentation pathway. In stark contrast, neither of the populations expressing HLA-II or the costimulatory molecules expanded in BLG-stimulated splenocytes from DR3 mice, despite appreciable increases in CD4^+^TCRβ^+^ and CD8^+^TCRβ^+^ subsets. Whether this means that BLG mediated responses raise anergy or tolerance in the DR3 genotype remains to be examined further. Interestingly, the levels of BLG-stimulated B cells that could act as cognate partners in eliciting T-cell mediated responses were also downregulated in DR3. Together, our *in vitro* studies uncovered potentially important cellular changes that might influence the pathophysiology of CMA in genetically distinct individuals.

Although HLA allele-dependent mechanisms that led to differential immune responses and symptoms were not directly examined in our present study, some determining factors may be speculated. First, the expansion of T cells with a distinct allergen-specific TCR Vβ repertoire may be differentially regulated by HLA-II alleles. Since the clonality of TCR Vβ, the variable domain of TCRβ chain, on CD4^+^ or CD8^+^ cells uniquely mediates tolerogenic or allergic responses (69), selective expansion of TCR Vβ subsets could have influenced symptomatic outcomes. Second, in addition to overriding effects of HLA-II allelic variations, other genes in proximity and linkage disequilibrium with HLA-II DR/DQ may exert a synergistic or antagonistic effect. For example, immune reactions to birch allergy are predominantly driven by TNFα polymorphisms rather than variations in HLA-II alleles (70). However, if certain HLA-II alleles also influence allergen-specific immune responses, they may have an additive effect on TNFα polymorphism, further altering symptom outcomes (70, 71). This type of link between HLA and TNF polymorphism has been described for chronic disease development, instead of spontaneous resolution, after hepatitis B infection (72). Finally, HLA allelic variants may differentially affect the extent of intestinal damage *via* distinct downstream immune responses, allowing transepithelial allergen entry. Subsequently, in addition to the variant-dependent presentation by APCs, infiltrated BLG may affect the brain and other organs by interfering with physiological function, particularly lipid and iron transport, given its structural and functional similarities to endogenous lipocalins (73). Investigating these possibilities may further clarify the distinct involvement of HLA-II alleles.

In summary, we used three HLA-II mouse strains and demonstrated that the variations in HLA-II alleles influenced CMA-associated symptom outcomes *via* unique immune signatures. However, the study was not without limitations and could be improved by the inclusion of naïve control groups and the assessment of allergy-associated cytokines and other serum factors at additional time points. Moreover, as with any experiments using humanized transgenic mice, it may be argued that the immune interactions between HLA-II, a human gene product expressed on mouse APCs, and TCR on mouse T cells are not physiological. Nevertheless, the role of HLA-II alleles in determining protective or severe outcomes by specifically potentiating T-cell responses has been demonstrated in these transgenic strains in an infection model (33), and the transgenic mouse strains provide valuable tools to test whether HLA-II alleles may potentially be considered as decisive or predictive factors for CMA development and particular symptom phenotypes.

## Conclusions

Our findings suggested that HLA-II alleles and sex contributed to variabilities in the severity of immediate physical symptoms, delayed behavioral manifestations, and systemic immune responses in our transgenic mouse model of CMA. The expression of DR3 may be linked to severe allergic symptoms *via* the classic type I hypersensitivity responses, while DQ8 may be associated with asymptomatic sensitization. Although DR15 is also linked to asymptomatic sensitization or mild symptoms, this allelic variant may predispose individuals to CMA-associated behavioral changes. Sex- and HLA-II-specific differences in allergen-induced immune responses were also demonstrated *in vitro*. Finally, the HLA-II transgenic mouse model of CMA provides an excellent tool to investigate the genetic influence on the disease development and symptom variations of food allergies that better reflect human conditions.

## Data Availability Statement

The data supporting the findings of this study are presented within the article or as Supplementary Materials. Further inquiries may be directed to the corresponding author.

## Ethics Statement

The animal study was reviewed and approved by University of North Dakota Institutional Animal Care and Use Committee.

## Author Contributions

KN-C: conceptualization, funding acquisition, project administration, resources, and supervision. DG, KN-C, and SN: data curation, visualization, investigation, methodology, validation, and writing—original draft preparation. KN-C, DG, SN, NS, and YW: formal analysis and writing—review and editing. All authors contributed to the article and approved the submitted version.

## Funding

Research reported in this publication was supported by NIH/NIGMS P20GM103442 and P20GM113123 and an institutional seed grant funded by the University of North Dakota Vice President of Research and Economic Development and School of Medicine & Health Sciences.

## Conflict of Interest

The authors declare that the research was conducted in the absence of any commercial or financial relationships that could be construed as a potential conflict of interest.

## Publisher's Note

All claims expressed in this article are solely those of the authors and do not necessarily represent those of their affiliated organizations, or those of the publisher, the editors and the reviewers. Any product that may be evaluated in this article, or claim that may be made by its manufacturer, is not guaranteed or endorsed by the publisher.
