# Supplementary figures and images for: HLA-II Alleles Influence Physical and Behavioral Responses to a Whey Allergen in a Transgenic Mouse Model of Cow's Milk Allergy
Source: Front Allergy. 2022 Apr 14;3:870513. doi: 10.3389/falgy.2022.870513 (PMC9234862; doi:10.3389/falgy.2022.870513)

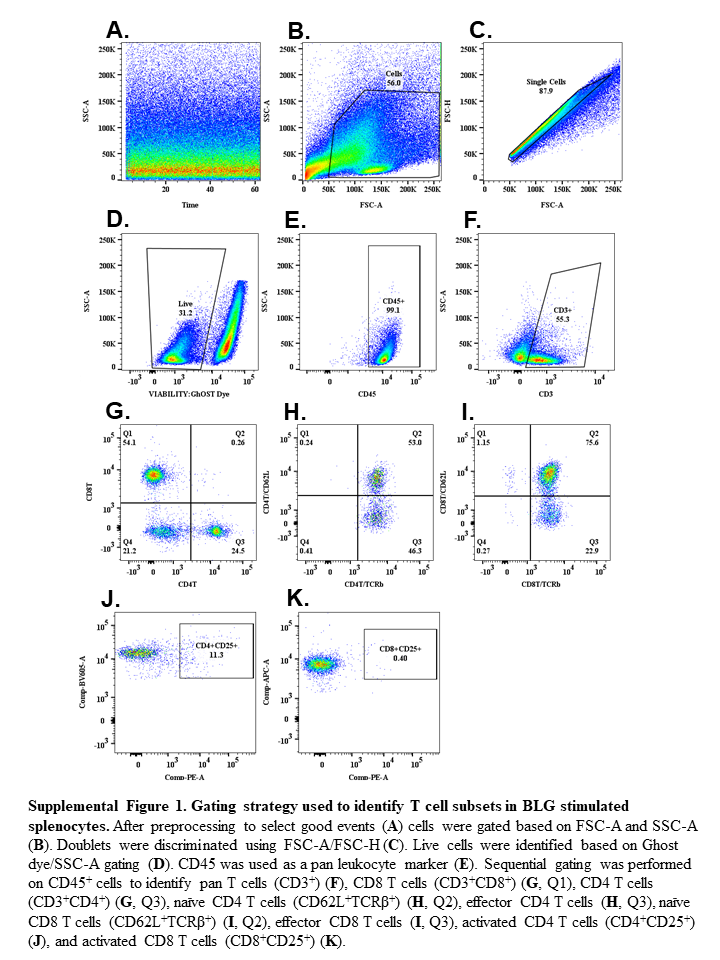

Supplement: Supplementary file 1 [file Image_1.TIF]

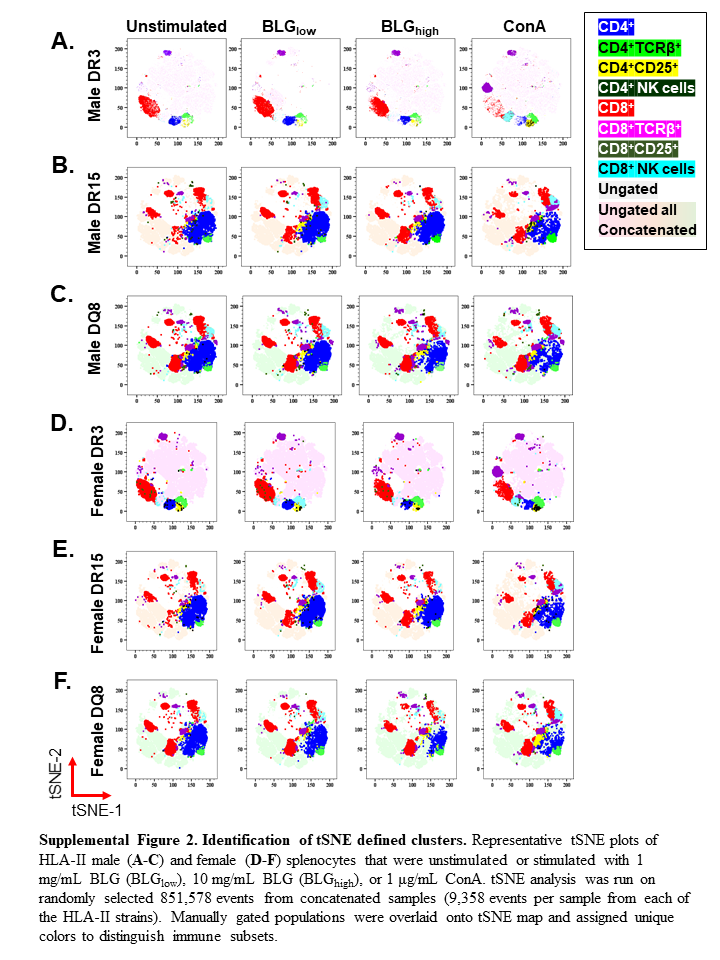

Supplement: Supplementary file 2 [file Image_2.TIF]

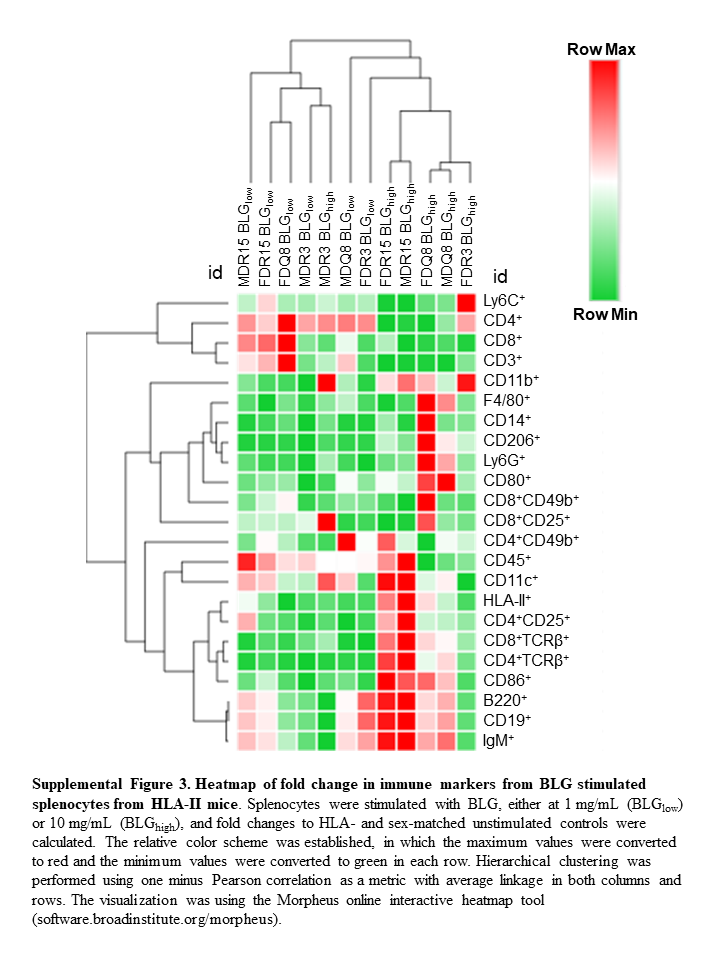

Supplement: Supplementary file 3 [file Image_3.TIF]

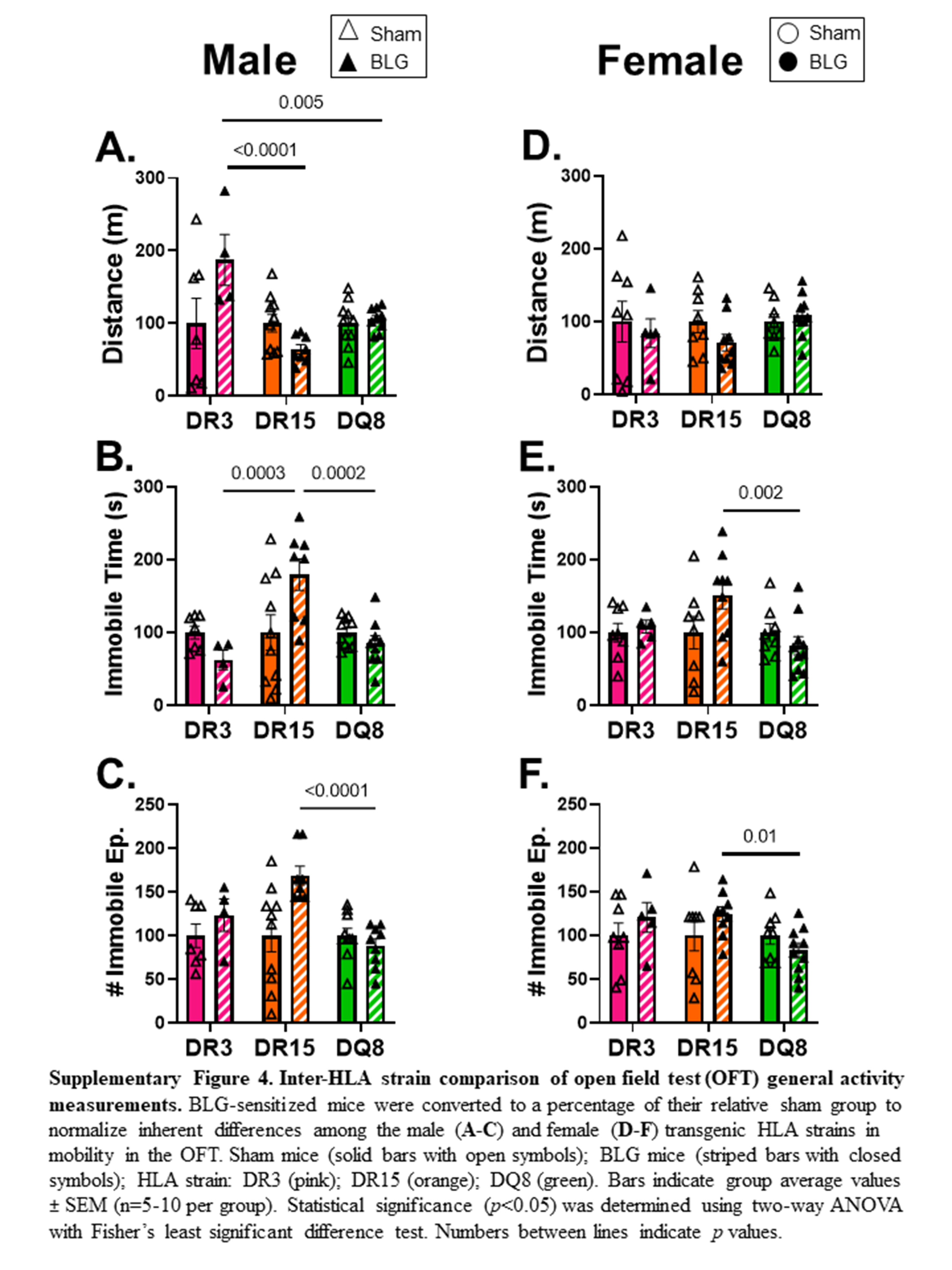

Supplement: Supplementary file 4 [file Image_4.TIF]

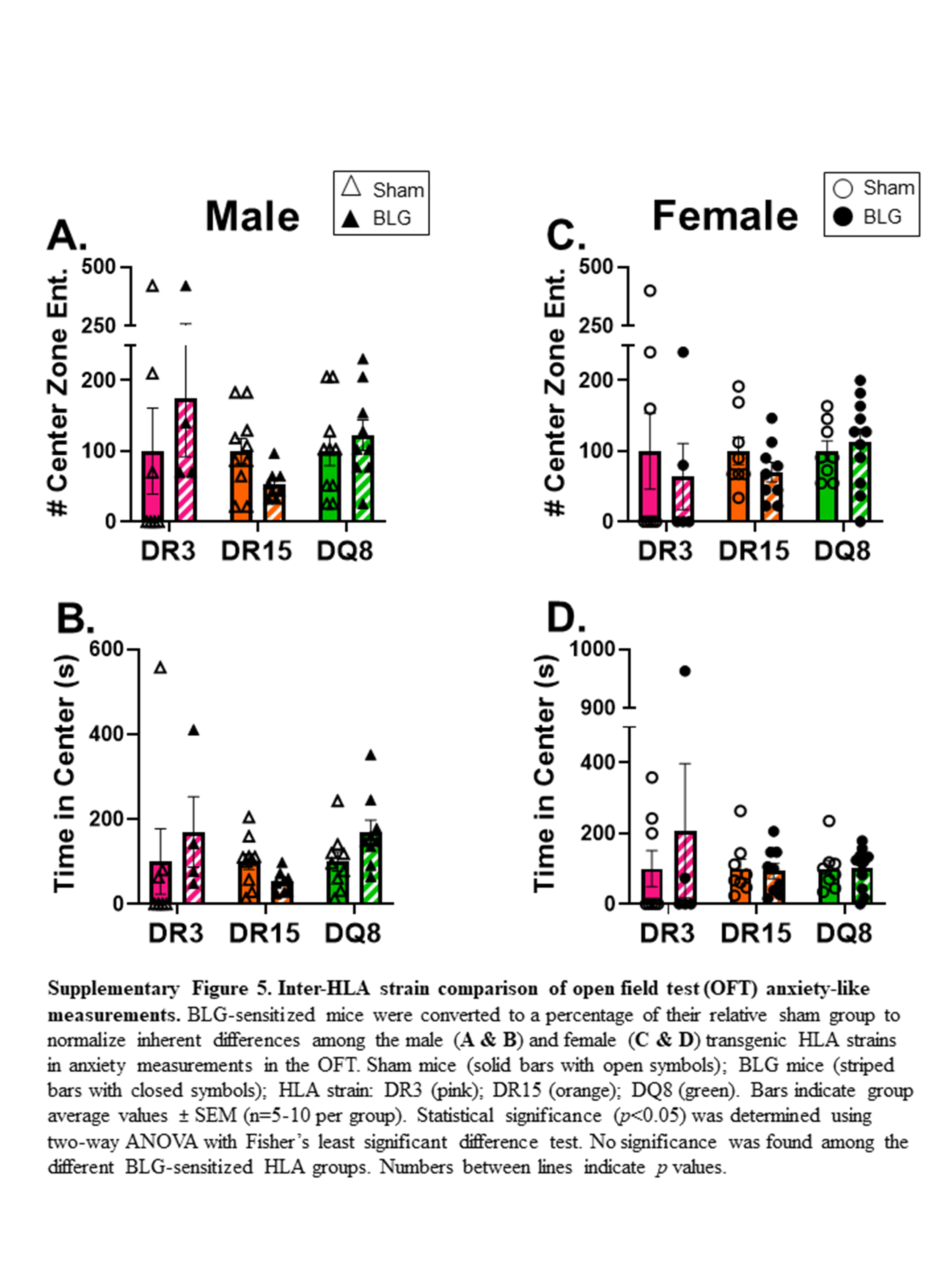

Supplement: Supplementary file 5 [file Image_5.TIF]

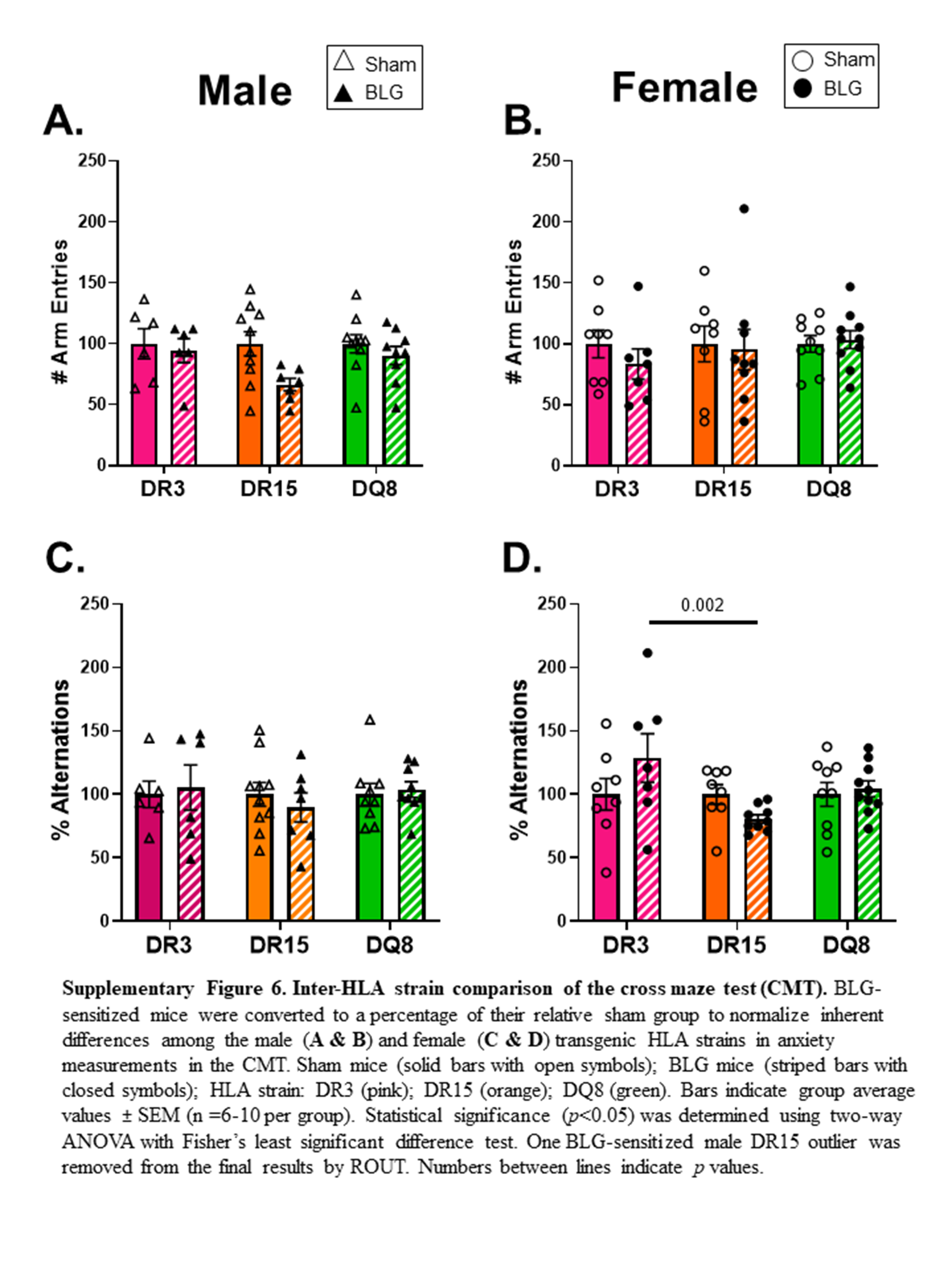

Supplement: Supplementary file 6 [file Image_6.TIF]

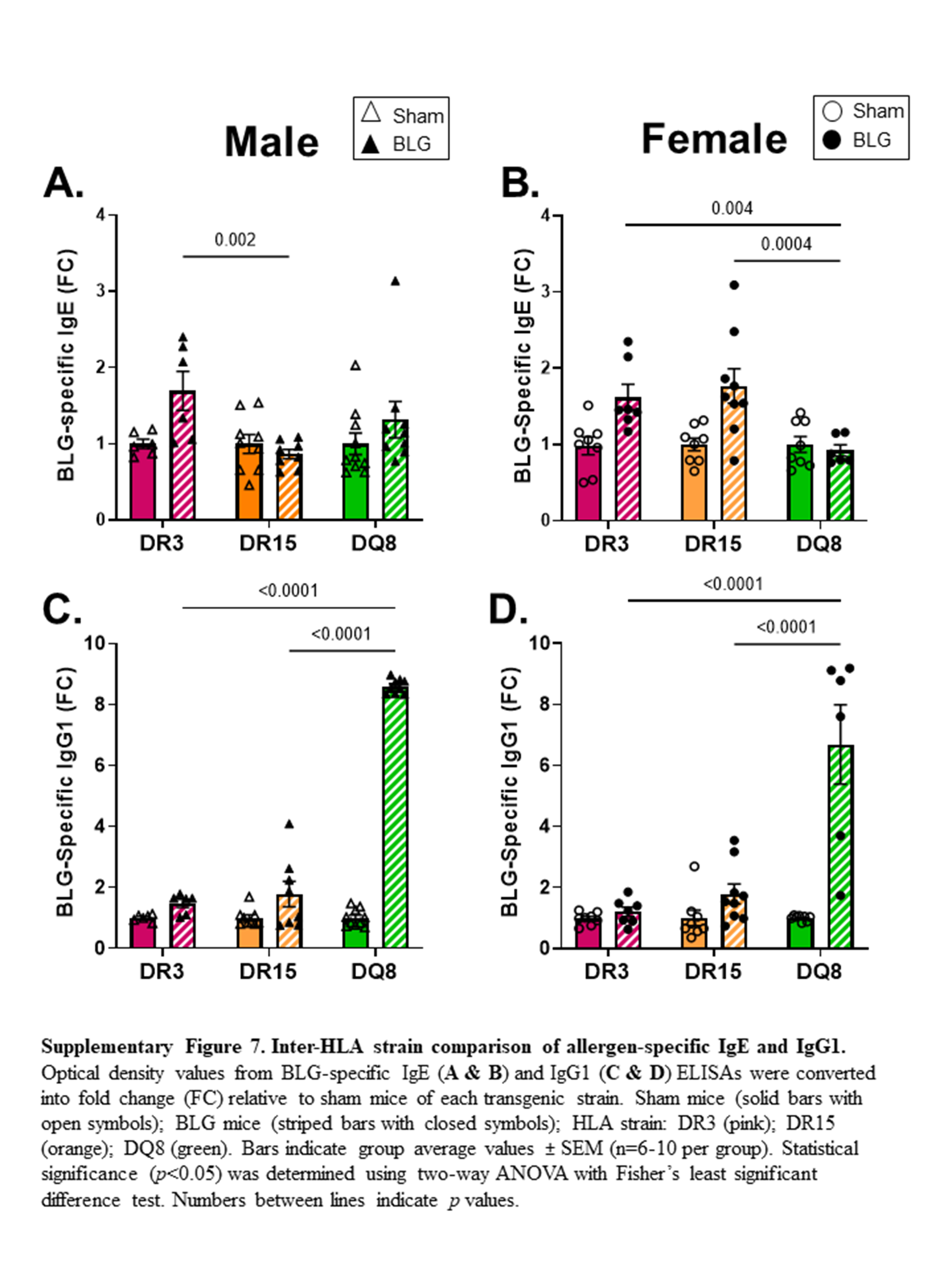

Supplement: Supplementary file 7 [file Image_7.TIF]

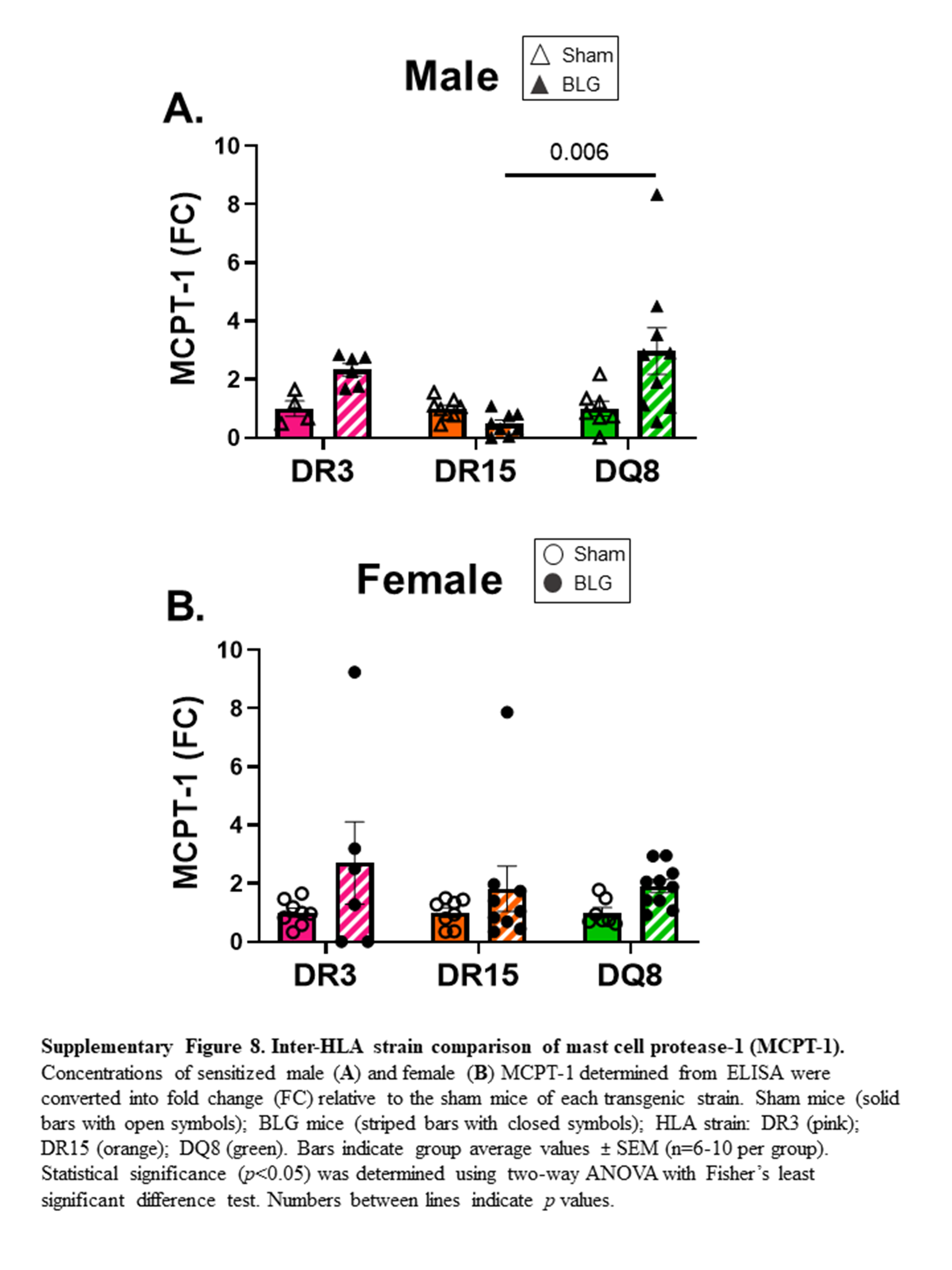

Supplement: Supplementary file 8 [file Image_8.TIF]
